# Acid sphingomyelinase activity suggests a new antipsychotic pharmaco-treatment strategy for schizophrenia

**DOI:** 10.1038/s41380-025-02893-6

**Published:** 2025-01-17

**Authors:** Daria Chestnykh, Christiane Mühle, Fabian Schumacher, Liubov S. Kalinichenko, Stefan Löber, Peter Gmeiner, Christian Alzheimer, Stephan von Hörsten, Burkhard Kleuser, Steffen Uebe, Arif B. Ekici, Erich Gulbins, Johannes Kornhuber, Hee Kyung Jin, Jae-sung Bae, Anbarasu Lourdusamy, Christian P. Müller

**Affiliations:** 1 Department of Psychiatry and Psychotherapy, University Clinic, Friedrich-Alexander-University of Erlangen-Nuremberg, Erlangen, Germany; 2 Institute of Pharmacy, Freie Universität Berlin, Berlin, Germany; 3 Department of Chemistry and Pharmacy, Medicinal Chemistry, Friedrich-Alexander-Universität Erlangen-Nürnberg, Nikolaus-Fiebiger-Str. 10, 91058 Erlangen, Germany; 4 FAUNeW—Research Center New Bioactive Compounds, Friedrich-Alexander-Universität Erlangen-Nürnberg, Nikolaus-Fiebiger-Str. 10, 91058 Erlangen, Germany; 5 Institute of Physiology and Pathophysiology, Friedrich-Alexander-University of Erlangen-Nuremberg, 91054 Erlangen, Germany; 6 Department of Experimental Therapy, Preclinical Experimental Center, Friedrich-Alexander-University of Erlangen-Nuremberg, Erlangen, Germany; 7 Institute of Human Genetics, Friedrich Alexander University of Erlangen-Nuremberg, Erlangen, Germany; 8 Department of Molecular Biology, University of Duisburg-Essen, 45147 Essen, Germany; 9 Department of Surgery, University of Cincinnati, College of Medicine, University of Cincinnati, Cincinnati, 231 Albert Sabin Way, Cincinnati, OH 45267-0558 USA; 10 KNU Alzheimer’s Disease Research Institute, Kyungpook National University, Daegu, 41566 South Korea; 11 Department of Laboratory Animal Medicine, College of Veterinary Medicine, Kyungpook National University, Daegu, 41566 South Korea; 12 Department of Physiology, School of Medicine, Kyungpook National University, Daegu, 41944 South Korea; 13 Academic Unit for Translational Medical Sciences, School of Medicine, University of Nottingham, Nottingham, NG7 2UH UK; 14 Department of Addictive Behavior and Addiction Medicine, Central Institute of Mental Health, Medical Faculty Mannheim, Heidelberg University, Mannheim, Germany

**Keywords:** Schizophrenia, Molecular biology

## Abstract

Schizophrenia is a chronic and severe mental disorder. It is currently treated with antipsychotic drugs (APD). However, APD’s work only in a limited number of patients and may have cognition impairing side effects. A growing body of evidence points out the potential involvement of abnormal sphingolipid metabolism in the pathophysiology of schizophrenia. Here, an analysis of human gene polymorphisms and brain gene expression in schizophrenia patients identified an association of *SMPD1* and *SMPD3* genes coding for acid- (ASM) and neutral sphingomyelinase-2 (NSM). In a rat model of psychosis using amphetamine hypersensitization, we found a locally restricted increase of ASM activity in the prefrontal cortex (PFC). Short-term haloperidol (HAL) treatment reversed behavioral symptoms and the ASM activity. A sphingolipidomic analysis confirmed an altered ceramide metabolism in the PFC during psychosis. Targeting enhanced ASM activity in a psychotic-like state with the ASM inhibitor KARI201 reversed psychotic like behavior and associated changes in the sphingolipidome. While effective HAL treatment led to locomotor decline and cognitive impairments, KARI201 did not. An RNA sequencing analysis of the PFC suggested a dysregulation of numerous schizophrenia related genes including Olig1, Fgfr1, Gpr17, Gna12, Abca2, Sox1, Dpm2, and Rab2a in the rat model of psychosis. HAL and KARI201 antipsychotic effects were associated with targeting expression of other schizophrenia associated genes like Col6a3, Slc22a8, and Bmal1, or Nr2f6a, respectively, but none affecting expression of sphingolipid regulating genes. Our data provide new insight into a potentially pathogenic mechanism of schizophrenia and suggest a new pharmaco-treatment strategy with reduced side effects.

## Introduction

Schizophrenia is a chronic and debilitating mental disorder that manifests through aberrations in thinking, perception, self-experience, cognitive functions, motivation, emotions, and behavior [1]. Both genetic and environmental factors are considered to play a crucial role in the etiology of the illness [2]. The dopamine (DA) and glutamate hypotheses of schizophrenia are the most influential ones, although inflammatory, synaptic, and membrane theories have been increasingly discussed lately [3, 4]. Antipsychotic drugs (APDs) are the foundation of the pharmacotherapy of schizophrenia and predominantly embody the antagonistic action of the DA D2 receptors in the meso-corticolimbic pathway [5, 6]. Despite the wide variety of available APDs, appropriate treatment still remains a challenge due to a loss of efficacy (treatment failure), drug resilience, devastating side effects, and even risk of mortality [7–10]. At present, antipsychotic medications primarily demonstrate efficacy in addressing positive symptoms, exhibiting minimal therapeutic benefit in alleviating negative and cognitive symptoms [11–13].

Based on early reports [14, 15], a growing body of evidence from recent neurolipidomic studies has suggested a potential role of sphingolipids in many psychiatric disorders, including schizophrenia. Sphingolipids critically shape synaptic structures in the nervous system as they conduct the formation of lipid rafts and clustering of receptors within it [16, 17]. They orchestrate intracellular signaling, neuronal connectivity, neuroinflammation response, proliferation, and apoptosis [18, 19]. Among them, ceramide- (Cer) and sphingomyelin (SM) species have been repeatedly linked to schizophrenia. A significant rise in Cer levels in the white matter has been observed in schizophrenic patients [20, 21]. Meanwhile, SM levels in red blood cell membranes and in the thalamus correlated negatively with the severity of psychotic symptoms and cognitive impairments of schizophrenics [22, 23]. Among the genetic polymorphisms associated with the illness, at least five genes are sphingolipid-related [24, 25]. These findings point to an involvement of sphingolipids and their enzyme rheostat in the pathogenesis of schizophrenia [26]. Here, we explore the implication of brain sphingolipid metabolism in the pathogenesis of schizophrenia, in mediating APD treatment efficacy and failure, and the possibility of using novel alternative modes of action for APD pharmacotherapy.

## Materials and methods

### Human association study

#### Genetic analysis

We obtained the Psychiatric Genomics Consortium (PGC) Wave 3 schizophrenia (SZ) genome-wide association study (GWAS) dataset from the PGC download page (https://pgc.unc.edu/for-researchers/download-results/). Informed consent was obtained from all subjects. This dataset is the summary statistics from the meta-analysis of core PGC-SZ samples of European and East Asian ancestry from the PGC, totaling 67,390 cases and 94,015 controls, and summary-level data from 7386 cases and 7008 controls from nine cohorts of African American and Latino ancestry. The PGC-SZ wave 3 dataset contains the odds ratios (ORs) and p-values that were calculated on more than 7 million SNPs in the meta-analysis of all cohorts. Regional visualization plots for four lipid genes were produced using LocusZoom.

#### Gene expression data analysis

The raw data was obtained from the Gene Expression Omnibus (GEO) database (GSE53987). This study contains samples of post-mortem brain tissue from control subjects and well-matched subjects with schizophrenia. RNA was isolated from Brodmann Area 46 (dorsolateral prefrontal cortex, DLPFC), associative subregion of dorsal striatum, and hippocampus, and gene expression profiles were generated using the Affymetrix Human Genome U133 Plus 2.0 arrays. Expression intensity values were calculated at probeset level for the 103 CEL files using the robust multi-array average (RMA) method. Probesets that are ‘absent’ (present/absent call using MAS5) in all samples were filtered out from the analysis. Expression values were mapped from probeset to unique gene and the probeset with the highest mean expression value was selected when multiple probesets were mapped to the same gene.

### Animal studies

#### Animals

We employed male Sprague–Dawley rats (N = 108), 8–9 weeks old, weighing 250-275 g at the arrival (Charles River, Germany). Male animals were used to avoid the potential hormonal biases in behavioral tests and based on the substantial sex differences in humans, where men display more severe clinical forms [27]. Rats were housed in groups of 3-4 per cage with food and water access *ad libitum*, in a temperature (22 ± 2 °C), humidity (55 ± 10%), and under a normal 12 h light–dark cycle (light on from 07:00). Behavioral tests were performed during the light phase.

#### Drugs and treatment procedures

First experiment in rats was performed in two batches with short- (N = 30) and long-term (N = 30) haloperidol (HAL) administration procedures. During the initial phase and after habituation, animals were sensitized with d-amphetamine (AMPH; Fagron) or 0.9% saline (SAL) in order to develop psychotic-like behavior as previously described [28, 29]. AMPH (1 ml/kg) was injected intraperitoneally (i.p.) three times a day for six consecutive days under the escalating dose regimen (from 1 to 8 mg/kg). Then, animals were implanted with osmotic mini-pumps (Alzet, DURECT Corporation) subcutaneously (s.c.) with 7-day (“2001” model, 200 µl, 1 µl/h) or 14-day (“2002” model, 200 µl, 0.5 µl/h) delivery of HAL (Sigma-Aldrich) or vehicle (VEH). HAL was mixed in distilled water, 0.3% ascorbic acid and 10% cyclodextrin, and VEH solution contained distilled water, 0.3% ascorbic acid and 10% cyclodextrin. The dose for HAL 0.5 mg/kg dose was used based on the clinically equivalent therapeutic dose and the occupancy level of the striatal D_2_ receptor at the therapeutic rate [30–32].

#### Implantation of osmotic mini-pumps

Surgeries were performed under the deep plane of anesthesia (ketamine 95 mg/ml + xylazine 12.5 mg/kg, i.p.) combined with systemic (carprofen, 5 mg/kg, s.c.) and local (Lidocaine, 0.1 ml) analgesia. An incision of 1.5-2 cm was made in each rat’s back, and a haemostat was applied to make a subcutaneous pocket. Mini-pumps were prepared one day before and incubated in sterile SAL at the room temperature overnight. During the surgery, they were placed in the subcutaneous pocket with the flow moderator directed away from the skin incision. The wound was closed with sterile 9 mm wound clips (Alzet, DURECT Corporation) and a tissue glue (VetBond). An antiseptic aluminum spray (Aluspray) was used to prevent local infections. After the surgeries and till the end of the experiment, rats were housed individually [30, 32]. For each batch, rats were assigned in a pseudorandomised manner into groups (n = 8–10/group): AMPH-sensitized HAL-treated (AMPH-HAL), AMPH-sensitized VEH-treated (AMPH-VEH), and SAL-sensitized VEH-treated (SAL-VEH).

#### ASM inhibition

The second experiment was designed to clarify the causal relationship between ASM activity and psychotic phenotype (N = 48) (Fig. 6a). Initially, we developed the psychotic-like behavior using the AMPH sensitization method as described in the first experiment. One day after the last AMPH injection, we started the treatment with oral gavage (8-cm curved stainless-steel gavage needles with 2.4-mm ball tip, CARL ROTH). Rats received HAL (1 mg/kg) or KARI201 (10 mg/kg) or VEH one time a day in the morning for ten days in total. During the testing days, drugs were administered one hour before a test for each rat. In this experiment, animals were distributed in four groups (n = 12/group): AMPH-HAL, AMPH-KARI, AMPH-VEH, and SAL-VEH. The ligand KARI201 was synthesized previously and demonstrated direct, selective, and competitive ASM inhibition with IC_50_ = 450 ± 1.58 nM. Pharmacokinetics studies showed high brain distribution, metabolic stability, and systemic bioavailability. KARI201 was found to have additional ghrelin receptor agonistic activity, and this dual action was considered beneficial in the amelioration of pathological symptoms in an Alzheimer’s disease mouse model [33].

#### Behavioral testing

To evaluate the manifestation of psychotic-like phenotype in rats, we carried out three behavioral tests: AMPH-induced hyperlocomotion (AIH), novel object recognition (NOR), and prepulse inhibition of acoustic startle (PPI) as described previously [29, 32].

##### Amphetamine-induced hyperlocomotion

AIH test has been commonly used in translational research to assess the efficacy of APDs. The method is based on a strong increase of locomotion, induced by a single low-dose injection of AMPH in previously sensitized animals [30]. The increased hyperlocomotion resembles the agitation status observed in schizophrenic patients and relates to positive, or psychotic, symptoms. Additionally, the anxiety level of animals was measured in the same test by calculating time spent, distance traveled and visits made in the central zone of the open field arena. This phenomenon is based on thigmotaxis, when rodents naturally prefer the periphery of the open arena. Decreased time spent in the central zone correlates with lower anxiety levels [34–36]. The enhanced anxiety has been considered a part of negative symptoms triad and was shown previously in several animal models of schizophrenia, including AMPH-induced psychotic model in rats [37–40].

The equipment was made from a square gray acrylic arena (45 × 45 × 45 cm H × L × W) and an illumination level of approximately 30 lx. The total area comprised the central zone (25 × 25 cm) and the peripheral zone (the rest of the area outside the central zone). A rat was placed in the corner of the arena for 20 min to record a baseline activity. Then an animal was removed and injected with AMPH (1.5 mg/kg, i.p.) and placed back into the arena for another 40 min. The activity was videotaped and analyzed by Biobserve Viewer III (Biobserve GmbH, Germany) or manually where appropriate.

##### Novel object recognition

NOR task serves as a tool to investigate the memory deficit in an animal model of schizophrenia. The paradigm refers to the exhibition of negative symptoms associated with cognitive impairment. Studies have shown the deficient short-term memory in animals after AMPH-induced psychosis [32]. NOR was carried out in the same gray acrylic arena that was used for the AIH testing as described above. We employed two pairs of objects: green metallic containers and white glass bottles, which were equivalent in size and counterbalanced between the groups. One-hour interval was set between test trials. First, each animal was exposed to two identical objects located in the opposite corners of the arena and allowed to freely explore them for 5 min. In the second trial, rats were placed back to the arena where one of the familiar objects was replaced with the new one. Again, rats were allowed to explore objects freely for 5 min. Biobserve Viewer III (Biobserve GmbH, Germany) was used for the video records. Behavioral analysis was performed manually with videotapes. Exploration behavior was defined as sniffing an object or touching it with the nose or paw [41, 42]. The discrimination rate was calculated with the following formula: Discrimination = ((Exploration time of the new object, sec) / (Exploration time of both new and familiar objects, sec)) × 100%.

##### Prepulse inhibition of acoustic startle

Sensorimotor gating deficit is a disruption recognized in human psychotic disorders and animal models of schizophrenia [43, 44], however, it was also reported in other psychiatric conditions. PPI assay is based on a principle where a low-intensity acoustic stimulus (prepulse or ppSt) can inhibit the startle reaction to a subsequent high-intensity acoustic stimulus (pulse or PSt). The PPI equipment (TSE Systems, Bad Homburg, Germany) represents soundproof boxes with small cages and speakers inside. The amplitude of startle response (ASR) was measured by piezoelectric accelerometers. Each session started from the placing rats in the individual restraining metal cages (27 × 9 × 10 cm) and then into the TSE boxes. Test trial was escorted by background noise (68 dB) and initiated with a 2-min adaptation period followed by PSt (100, 110 and 120 dB, twice for each intensity). After that, 16 pseudorandomized trials were transmitted ten times. The further session consisted of three ppSt (74, 80, 86 dB), three PSt (100, 110 and 120 dB), nine combinations of ppSt + PSt, and a no stimulus trial. At the end, six PSt were played again. The duration of acoustic stimuli was 20 ms and 30 ms, for ppSt and PSt respectively. In the combination pp + P stimulus, the intratrial delay was set at 100 ms. The interval between trials was defined at 15 s [45, 46]. The magnitude of startle response was registered automatically by TSE Systems. The PPI percentage was calculated manually with the formula: %PPI = 100 – [100 x (ppSt + PSt ASR amplitude) / (PSt ASR amplitude)] [28, 30, 32]. The area under the curve (AUC) was computed by trapezoidal integration and ROC function using SPSS software. The individual AUC values were calculated based on the curves for each combination of PSt with three ppSt (see Figs. S1d–f and S2e–g). Data were presented graphically as mean AUC ± SEM for each experimental group.

#### Sphingolipid-metabolizing enzymes activities

The activity of sphingolipid metabolizing enzymes in the brain tissue was determined using the fluorescently labeled substrates BODIPY-FL-C12-SM (Invitrogen, Thermo Fisher Scientific, USA) for sphingomyelinases, NBD-C12-ceramide (Cayman, obtained from Biomol, Hamburg, Germany) for ceramidases, C6-NBD-ceramide (Cayman, obtained from Biomol, Hamburg, Germany) for sphingomyelinase synthase with four replicates for each sample according to the previously described protocol [47]. First, thawed brain tissues were homogenized in sucrose lysis buffer (450 µl) comprising of 250 mM sucrose, 1 mM EDTA, and 1x complete tablets EASY pack protease inhibitor cocktail (Roche) in a Tissue Lyser LT bead mill (Qiagen, Hilden, Germany) with steal beads followed by ultrasound exposure for 1 min in a water bath. Homogenization was enhanced by one freeze-thaw cycle and additional sonication for 2 min after transfer of the lysates to a 96-well-polystyrene plate. For the assay, homogenized tissues were diluted at the ratio 1:16 by the addition of NaCl (154 nM). Protein concentrations were quantified using the Pierce™ Coomassie Plus (Bradford) Assay Reagent (Thermo Fisher Scientific, Waltham, MA, USA) and on a ClarioStar Plus microplate reader (BMG Labtech, Germany). For the enzymatic reaction, the reaction mix contained fluorescently labeled substrate (25 pmol) in reaction buffer with 200 mM HEPES buffer (pH 7.35), 200 mM MgCl_2_, 0.05% IGEPAL® CA-630 (NP 40) for NSM, 200 mM sodium acetate buffer (pH 5.0), 500 mM NaCl, 0.2% IGEPAL® CA-630 (NP 40) detergent for ASM, 200 mM sodium acetate buffer (pH 4.5), 100 mM NaCl, 0.03% IGEPAL® CA-630 (NP 40) for AC; 200 mM HEPES (pH 7.35), 100 mM NaCl, 0.03% IGEPAL® CA-630 (NP 40) for NC; and 50 mM Tris-HCl (pH 7.4), 25 mM KCl, 0.5 mM EDTA (pH 8.0), and 0.1 mM phosphatidylcholine for SMS. The reaction was initiated by the addition of 8 µl (0.5–1 µg protein) of tissue lysate in 96-well polystyrene plates. After incubation at 37 °C for 0.3–24 h (150 min – ASM, 20 min – NSM, 195 min – AC, 90 min – NC, 24 h - SMS) reactions were stopped by freezing at −20 °C and stored until further procedures. For the substrate and product separation, 1.5 µl of each sample was directly spotted on silica gel 60 thin-layer chromatography plates (ALUGRAM SIL G, Macherey-Nagel, Dueren, Germany), for SMS on chloroform/methanol/ ammonium hydroxide (25% NH3 (aq)) /water (70:30:4:1, v/v/v/v). After 20 min of drying, the plates were immersed into the solvent (1% acetic acid in ethyl acetate). The fluorescent signal was detected in a Typhoon Trio scanner (488 nm excitation, 520 nm emission, 325 − 385 V, 1200  µm resolution, GE Healthcare Life Sciences, Buckinghamshire, UK) and quantified with the ImageQuant software (GE Healthcare Life Sciences, Buckinghamshire, UK). The activity of enzymes was estimated based on a conversion ratio between sphingomyelin and ceramide per 1 h and per 1 µg of protein.

#### Sphingolipid species measurement

The analysis was performed using liquid chromatography coupled to tandem-mass spectrometry (LC-MS/MS) as described previously [48, 49]. Lipids were extracted from aqueous brain tissue homogenates using a mixture of 1.5 ml methanol and chloroform in a 2:1 volume ratio. The extraction solvent contained internal standards, including C17:0 ceramide (Cer 17:0), and d31-C16:0 sphingomyelin (d31-SM 16:0) from Avanti Polar Lipids, Alabaster, USA. Chromatographic separations were achieved on an Agilent Technologies (Waldbronn, Germany) 1290 Infinity II HPLC system equipped with a Poroshell 120 EC-C8 column (3.0 × 150 mm, 2.7 µm). The MS/MS analyses were carried out using an Agilent Technologies 6495 C triple-quadrupole mass spectrometer operating in the positive electrospray ionization mode (ESI + ). The mass transitions recorded for various lipids and their qualifier product ions in parentheses were as follows: m/z 520.5 → 264.3 (282.3) for Cer 16:0, m/z 534.5 → 264.3 (282.3) for Cer 17:0, m/z 548.5 → 264.3 (282.3) for Cer 18:0, m/z 576.6 → 264.3 (282.3) for Cer 20:0, m/z 604.6 → 264.3 (282.3) for Cer 22:0, m/z 630.6 → 264.3 (282.3) for Cer 24:1, m/z 632.6 → 264.3 (282.3) for Cer 24:0, m/z 703.6 → 184.1 (86.1) for SM 16:0, m/z 731.6 → 184.1 (86.1) for SM 18:0, m/z 734.6 → 184.1 (86.1) for d31-SM 16:0, m/z 759.6 → 184.1 (86.1) for SM 20:0, m/z 787.7 → 184.1 (86.1) for SM 22:0, m/z 813.7 → 184.1 (86.1) for SM 24:1, and m/z 815.7 → 184.1 (86.1) for SM 24:0. Peak areas of ceramide and sphingomyelin subspecies, as determined with MassHunter software (version 10.1, Agilent Technologies), were normalized to those of the internal standards followed by external calibration in the range of 1 fmol to 50 pmol on column. Determined sphingolipid amounts were normalized to the actual protein content (as determined by Bradford assay) of the tissue homogenate used for extraction (pmol/mg protein).

#### RNA-Seq analysis in rats

AMPH-sensitized HAL-treated (AMPH-HAL), AMPH-sensitized KARI201-treated (AMPH-KARI), AMPH-sensitized VEH-treated (AMPH-VEH), and SAL-sensitized VEH-treated (SAL-VEH) rats were sacrificed after behavioral testing and prefrontal cortex(PFC) tissue collected. RNA was isolated using the RNeasy Micro Kit (Qiagen). Sequencing libraries were generated from 1 µg high quality RNA using the Illumina Stranded mRNA Library Prep Kit (Illumina, San Diego, U.S.A.). Libraries were sequenced on a NovaSeq-6000 platform (Illumina, San Diego, U.S.A.). Differentially expressed genes were determined using a negative binomial model (Supplementary Methods).

#### Statistical analysis

The data are displayed as means ± standard error of the mean (SEM). Data were analyzed with IBM SPSS Statistics software (version 28). Our statistical null hypothesis was to compare the effects of treatment after AMPH sensitization with controls. We applied one- or two-way analysis of variance (ANOVA) to reveal the effects of factors and their interaction. Pre-planned comparisons with Bonferroni-corrected least significant difference (LSD) test were exploited to indicate the significant differences between the groups. The significance level was set at p < 0.05.

## Results

### Association of *SMPD1* and *SMPD3* with schizophrenia in humans

**Fig. 1:**
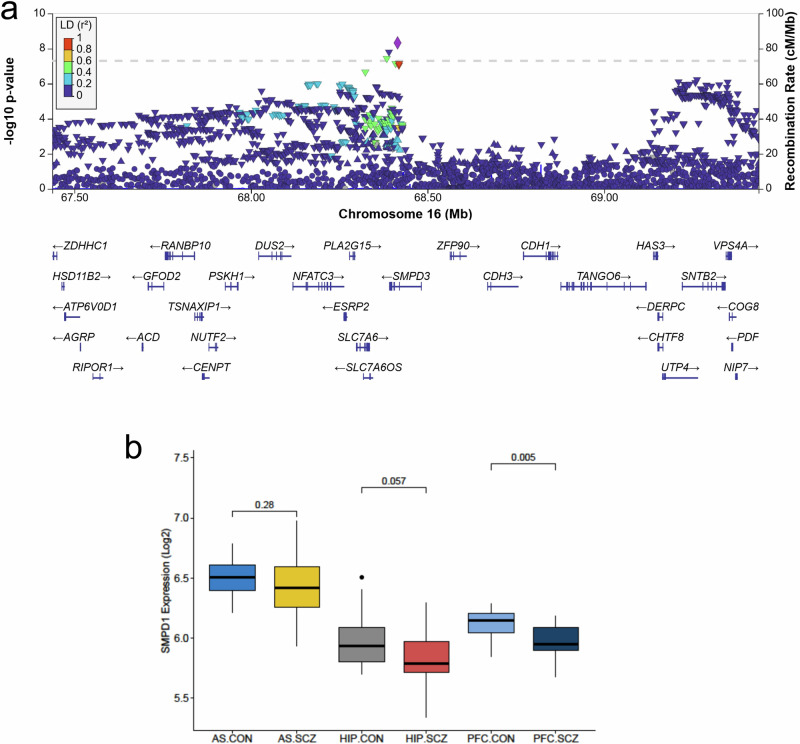
Association of genes coding for sphingolipid regulatory enzymes neutral sphingomyelinase (NSM) and acid sphingomyelinase (ASM) with schizophrenia in humans. **a** LocusZoom plot cantered on *SMPD3* gene for the Schizophrenia genome-wide association study. In this plot, each point is one variant, with the *x*- and *y*-axes representing physical position on the chromosome and −log_10_(p-value), respectively. The lead SNP (rs11862968; p-value = 4.72 × 10^−9^) is represented by the purple diamond symbol. The color coding of all other SNPs indicates LD with the lead SNP (estimated by Phase II HapMap CEU r^2^ values): red, r^2^ ≥ 0.8; gold, 0.6 ≤ r^2^ < 0.8; green, 0.4 ≤ r^2^ < 0.6; cyan, 0.2 ≤ r^2^ < 0.4; blue, r^2^ < 0.2; gray, r^2^ unknown. Recombination rates are estimated from 1000 Genomes phase 3 data. The dashed horizontal line represents the statistical threshold of genome-wide significance (−log_10_ (P = 5 × 10^−8^)). **b** Comparisons of *SMPD1* gene expression values in each of the three-brain region. The normalized (log2) expression values of SMPD1 gene were compared between disease states (CON: Control and SCZ: Schizophrenia) in Associative Striatum (AS): CON = 18 and SCZ = 18, Hippocampus (HIP): CON = 18 and SCZ = 15, and Pre-frontal Cortex (PFC): CON = 19 and SCZ = 15 using the Wilcoxon test. In the box plots, the box represents the interquartile range (IQR), with the upper and lower bounds indicating the 75th percentile (Q3) and 25th percentile (Q1) respectively. The horizontal line inside the box represents the median. The upper and lower horizontal lines outside the box indicate the maximum and minimum values (excluding outliers), which correspond to Q3 + 1.5 × IQR and Q1 − 1.5 × IQR respectively. The whiskers extend from each quartile to the minimum and maximum values.

In order to test whether natural genetic variance in genes of sphingolipid control predisposes to schizophrenia, we performed a human genome-wide association study. For four key sphingolipid regulatory genes involved in cognitive and emotional behavioral regulation [50, 51], the major two related to ceramide synthesis (*SMPD1* and *SMPD3)* and major two related to ceramide metabolism (*ASAH1* and *ASAH2*), we extracted single-nucleotide polymorphisms (SNPs) that cover a gene region (extended to ± 10 KB) and their association with schizophrenia from the latest genome-wide association study (GWAS) summary statistics of wave 3 Psychiatric Genomics Consortium (PGC) GWAS of schizophrenia. It included 74,776 cases and 101,023 controls [25]. Among the four analyzed sphingolipid-genes, we identified three SNPs in *SMPD3*, coding for neutral sphingomyelinase-2 (NSM), that showed significant association with schizophrenia (Fig. 1a). No associations were found for *SMPD1, ASAH1* and *ASAH2*.

Next, we compared the normalized expression values of these sphingolipid controlling genes between controls (CON) and schizophrenia (SCZ) patients in three different brain regions: the associate striatum (AS: n_CON_ = 18 and n_SCZ_ = 18), hippocampus (HIP: n_CON_ = 18 and n_SCZ_ = 15), and the prefrontal cortex (PFC: n_CON_ = 19 and n_SCZ_ = 15) [52]. Among four sphingolipid genes, we identified significant expression alterations for *SMPD1*, coding for ASM, specifically in the PFC (log_2_ Fold change = −0.14; P = 0.005, Wilcoxon test; Fig. 1b). Altogether, these findings point towards an association of genes coding for NSM and ASM with schizophrenia, with the PFC as a potential locus of action.

### Psychosis-like behavior can be reversed by short-term haloperidol treatment

**Fig. 2:**
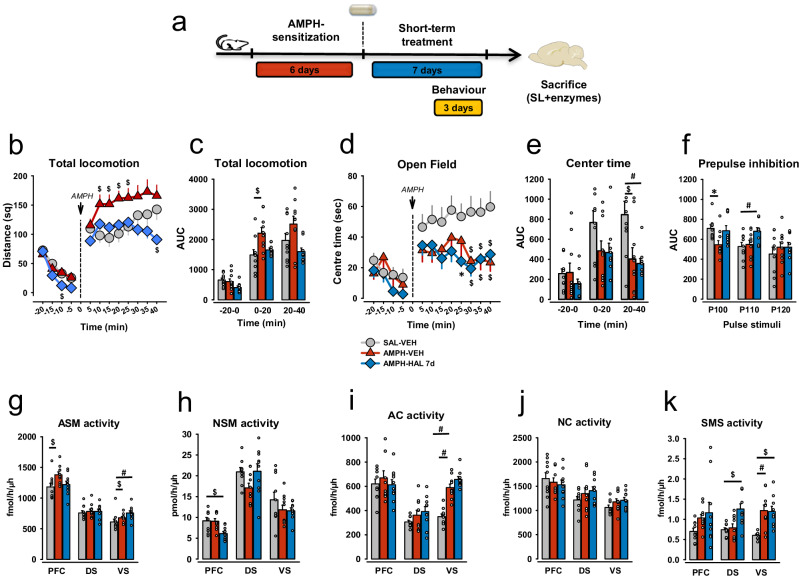
Experimental designs and behavioral effects of short-term chronic treatment with haloperidol and impact on the activity of brain acid sphingomyelinase. Data are presented as means ± SEM. **a** Study design for the short-term (7 days of treatment) experiment. **b** Total locomotion of animals in AMPH-induced hyperlocomotion (AIH) test with short-term HAL treatment (7 days of osmotic mini-pumps delivery, 0.5 mg/kg). AMPH-sensitized rats treated with vehicle (AMPH-VEH) showed a psychotic-like agitation state by the dramatically increased locomotion after the AMPH challenge (1.5 mg/kg, i.p.) in comparison with HAL-treated (AMPH-HAL) and control (SAL-VEH) groups. **c** Area under the curve (AUC)for total locomotion level after short-term HAL treatment (7 days, 0.5 mg/kg). **d** Time spent in the central zone of the open field in the AIH test after short-term HAL treatment. **e** AUC for time spent in the central zone of the open field in the AIH test after short-term HAL treatment. **f** AUC for prepulse inhibition (PPI) of acoustic startle response for three pulse stimuli: 100, 110, and 120 dB after short-term HAL treatment. Rats with psychotic-like symptoms demonstrate lower AUC of PPI and thus higher sensorimotor gating deficit that was restored by short-term treatment with HAL. **g** Activity of acid sphingomyelinase (ASM) after short-term HAL treatment in three brain regions: prefrontal cortex (PFC), dorsal (DS) and ventral striatum (VS). **h** Neutral sphingomyelinase (NSM) activity in three brain regions: PFC, DS, and VS after short-term HAL treatment. **i** Acid ceramidase (AC) activity in three brain regions: PFC, DS, and VS after short-term HAL treatment. **j** Neutral ceramidase (NC) activity in three brain regions: PFC, DS, and VS after short-term HAL treatment. **k** Sphingomyelin synthase (SMS) activity in three brain regions: PFC, DS, and VS after short-term HAL treatment. (*p < 0.05, ^#^p < 0.01, ^$^p < 0.001; n = 8–10 animals/group).

After principle relevance of sphingolipid genes in schizophrenia was identified, we searched for brain mechanisms of sphingolipid involvement in schizophrenia pathogenesis and in APD therapy. To interrogate the role of sphingolipid regulatory enzymes in schizophrenia, we investigated behavior in a rat model of an amphetamine (AMPH)-induced psychosis [28, 53] after short-term treatment with haloperidol (HAL) [30, 54]. Escalating AMPH-treatment induced psychosis-like behavior in a test of AMPH-induced acute hyperlocomotion (AIH) and in the pre-pulse inhibition of an acoustic startle response (PPI). A seven-day short-term chronic administration of HAL with osmotic mini-pumps (Figs. 2a and S1) improved psychotic-like symptoms of previously AMPH-sensitized rats in the AIH test and PPI test. These two tests are commonly used in preclinical assessment of novel APDs and correspond to agitation behavior and sensorimotor gating deficits of schizophrenic patients. In detail, for the short-term treatment model, two-way ANOVAs showed significant effects for the factor *Group* in the baseline (F_2,104_ = 6.374, p = 0.002) and challenge period (F_2,208_ = 29.562, p < 0.001) of the total locomotion level in the AIH test (Fig. 2b). The factor *Time* yielded a significant effect during baseline (F_3,104_ = 40.005, p < 0.001), but only a tendency after the AMPH challenge (F_7,208_ = 1.923, p = 0.067). No significant effects of *Time×Group* interaction were observed (F_6,104_ = 1.112, p = 0.361 and F_14,208_ = 1.212, p = 0.268), before and after AMPH injection. AMPH-sensitized vehicle-treated animals (AMPH-VEH) displayed a longer distance moved in the open field than a control group (SAL-VEH) at 10-, 15-, 20-, and 25-min time points after the AMPH challenge (p = 0.005, p = 0.002, p = 0.002, p = 0.009, respectively, Fig. 2b). AMPH-sensitized HAL-treated animals (AMPH-HAL) showed significantly lower locomotion then controls at the baseline and after AMPH challenge (p = 0.012 and p = 0.008 at −10 min and 25 min time points). An area under the curve (AUC) analysis with two-way ANOVA with LSD pre-planned comparisons verified the higher locomotion level for the AMPH-VEH group compared to controls (p = 0.002 for the first 20 min after AMPH injection), which was reversed by HAL treatment (Fig. 2c).

The anxiety level of animals was evaluated by the time spent in the center area of the Open field (OF). Animals from both AMPH-sensitized groups displayed higher anxiety levels after the AMPH challenge, indicated by a more prominent decline during the second 20 min of testing (Fig. 2d). There were significant effects of the factors *Time* (F_2,78_ = 11.973, p < 0.001) and *Group* (F_2,78_ = 8.194, p < 0.001), but not for the *Interaction* (F_4,78_ = 1.760, p = 0.145). AUC analysis with pre-planned comparisons showed significant differences between AMPH-VEH (p = 0.001) and AMPH-HAL (p < 0.001) groups vs. controls (Fig. 2e).

We found sensorimotor gating deficit in the PPI test in AMPH-VEH rats (p = 0.014 vs SAL-VEH) for the P100 dB stimulus (Factor: *Prepulse stimulus (ppSt)*, F_2,75_ = 39.278, p < 0.001; Factor: *Group*, F_2,75_ = 3.751, p = 0.028; *Interaction*, F_4,75_ = 3.751, p = 0.594, Fig. 2f). For P110 dB, we found an increase of the PPI AUC in the AMPH-HAL group (p < 0.001 vs SAL-VEH; two-way ANOVA, Factor: *ppSt*, F_2,75_ = 89.632, p < 0.001, Factor *Group*, F_2,75_ = 6.686, p = 0.002, *Interaction*, F_4,75_ = 1.657, p = 0.169) (Fig. 2f). These findings replicate the previously established schizophrenia model along the sensomotor-, emotional- and cognitive dimensions of the pathology.

### Amphetamine-induced psychotic-like behavior is associated with sphingolipid enzyme activity changes

In order to identify the sphingolipid neurobiology underlying schizophrenia pathogenesis and treatment outcomes, we measured the activity of five sphingolipid-metabolizing enzymes: ASM, NSM, acid- (AC) and neutral (NC) ceramidases, as well as sphingomyelin synthase (SMS) in three different brain regions most related to schizophrenia pathogenesis: the PFC, which is mostly associated with cognitive and negative symptoms, and the dorsal- (DS) and ventral striatum (VS) that are predominantly claimed to be responsible for psychotic symptoms [3]. After psychosis-induction, we observed a significantly enhanced ASM activity in the PFC (F_2,26_ = 6.387, p = 0.006; AMPH-VEH vs Sal-VEH: p = 0.002) and VS (F_2,25_ = 12.697, p < 0.001, AMPH-VEH vs Sal-VEH: p = 0.002). Also, in the VS, AC activity (F_2,26_ = 40.821, p < 0.001; p < 0.001) and SMS activity (F_2,25_ = 9.205, p = 0.001; p < 0.001) were significantly enhanced in these animals (Fig. 2g–k). This may suggest a potential pathological mechanism linking psychosis-induction and sphingolipid metabolism. Sphingolipid changes coincide with the observation of psychotic-like behavioral symptoms.

### Psychosis-remission is associated with normalization of sphingolipid enzyme activity

If a patho-mechanism is associated with both, psychosis-induction and APD treatment efficacy, it should emerge during psychosis induction, but disappear when APD treatment is effective. Effective short-term treatment with HAL did not impact the psychosis-induced rise in the activity of the enzymes, except ASM in the PFC. ASM activity was restored to the levels of the SAL-VEH group after 7-day administration with HAL, mirroring the efficacy pattern at behavioral level (Fig. 2g). In addition, HAL treatment attenuated NSM activity in the PFC (p p < 0.001). Short-term HAL treatment did not affect the AMPH sensitization induced increase in VS ASM (p < 0.001; Fig. 2g), AC (p < 0.001; Fig. 2i) and SMS activity (p < p < 0.001; Fig. 2k). These findings may suggest a specific role for ASM in the PFC for psychosis induction and reversal.

### Brain sphingolipids during psychosis-induction and reversal

**Fig. 3:**
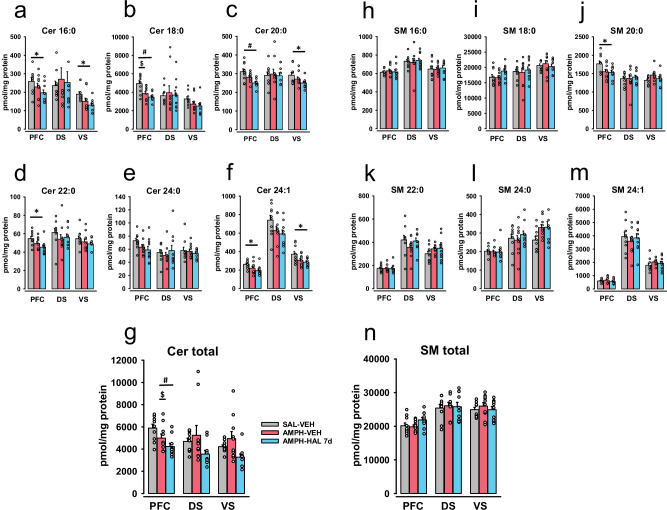
The concentrations of ceramide and sphingomyelin species after short-term (7 days) haloperidol treatment in three brain structures: PFC, DS and VS. Data are presented as means ± SEM. **a** Levels of ceramide 16:0 (Cer 16:0). **b** Levels of ceramide 18:0 (Cer 18:0). **c** Levels of ceramide 20:0 (Cer 20:0). **d** Levels of ceramide 22:0 (Cer 22:0). **e** Levels of ceramide 24:0 (Cer 24:0). **f** Levels of ceramide 24:1 (Cer 24:1). **g** Levels of total ceramide (Cer Total). **h** Levels of sphingomyelin 16:0 (SM 16:0). **i** Levels of sphingomyelin 18:0 (SM 18:0). **j** Levels of sphingomyelin 20:0 (SM 20:0). **k** Levels of sphingomyelin 22:0 (SM 22:0). **l** Levels of sphingomyelin 24:0 (SM 24:0). **m** Levels of sphingomyelin 24:1 (SM 24:1). **n** Levels of total sphingomyelin (SM Total). Data were analyzed by one-way ANOVA followed by LSD pre-planned comparisons with Bonferroni’s correction (n = 8–10 animals/group; *p < 0.05, ^#^p < 0.01, ^$^p < 0.001).

Sphingolipid enzymes tightly regulate ceramide and SM levels in a brain-area specific way [16, 26, 50]. In order to detect potential patterns of sphingolipids that may be associated with psychosis induction and reversal, we measured single species and total ceramide and SM levels in the PFC, VS and DS. We found a decline in most of the analyzed ceramide species in the PFC and some species in the VS in AMPH-sensitized animals, regardless of the treatment (Fig. 3a–f). This was reflected in a significant decline in total ceramide levels in the PFC (F_2,27_ = 9.862, p < 0.001; AMPH-VEH and AMPH-HAL vs. SAL-VEH: p = 0.005 and p < 0.001), but not in VS or DS (p > 0.05) (Fig. 3g).

Surprisingly, the remission of psychosis–like behavior with HAL was not accompanied by reversal of ceramide alterations. In fact, the visually detectable decline was now significant in the PFC for Cer 16:0 (p = 0.024), Cer 20:0 (p < 0.001), Cer 22:0 (p = 0.005), and Cer 24:1 (p = 0.009) in the AMPH-HAL group compared to controls. Also the most abundant Cer 18:0 further declined in the PFC (p < 0.001), driving the decline in total ceramide levels (p < 0.001). Furthermore, Cer 16:0, Cer 20:0 and Cer 24:1 levels were found to be significantly lower in the VS of AMPH-HAL rats compared to controls (p = 0.006, p = 0.017, p = 0.008). The treatment had only little effects on SM levels in the PFC, VS or DS (Fig. 3h–n). Only SM20:0 levels were reduced in the PFC after AMPH-HAL treatment (p = 0.018; Figs. 3j and S2). Altogether, these findings suggest PFC and VS specific alterations of Cer levels following psychosis induction. However, they may not be directly involved in the APD treatment response as they do not reverse after HAL short-term chronic treatment.

### AMPH-induced psychosis is diminished after 14 days

**Fig. 4:**
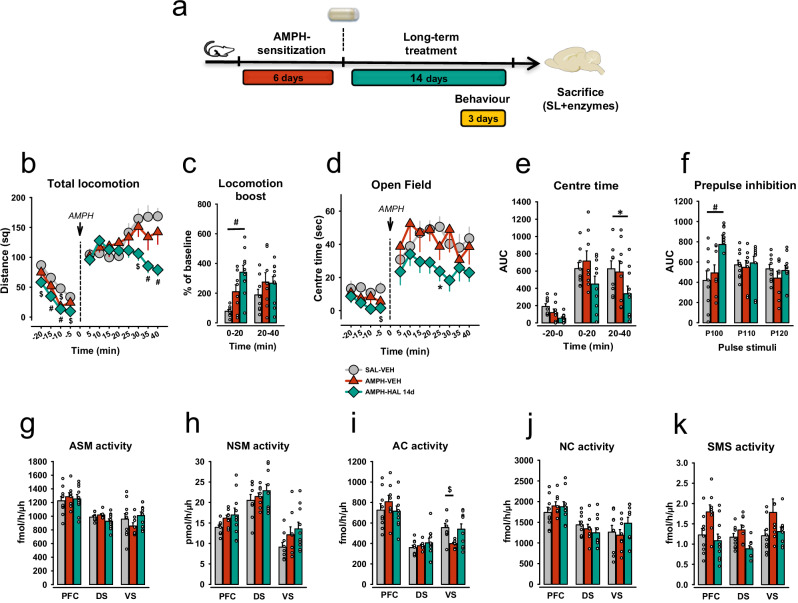
Experimental designs and behavioral effects of long-term chronic treatment with haloperidol and impact on brain activity of acid sphingomyelinase. Data are presented as means ± SEM. **a** Study design for the long-term (14 days of treatment) experiment. **b** Total locomotion of animals in AMPH-induced hyperlocomotion (AIH) test with long-term HAL treatment (14 days, 0.5 mg/kg). **c** Locomotion boost between baseline level and after AMPH challenge for two 20-min intervals after long-term HAL treatment (14 days, 0,5 mg/kg). **d** Time spent in the central zone of the open field in the AIH test after long-term HAL treatment. (p) AUC for time spent in the central zone of the open field in the AIH test after long-term HAL treatment. **e** The discrimination level between novel and familiar objects in the novel object recognition (NOR) test after long-term HAL treatment. AMPH-sensitized animals display a deficit in short-term memory resulting in a lower discrimination rate. **f** AUC for prepulse inhibition (PPI) of acoustic startle for three pulse stimuli 100, 110, and 120 dB after long-term HAL treatment. **g** The activity of acid sphingomyelinase (ASM) after long-term HAL treatment in the PFC, DS, and VS. **h** NSM activity in three brain regions: PFC, DS, and VS after long-term HAL treatment. **i** AC activity in three brain regions: PFC, DS, and VS after long-term HAL treatment. **j** NC activity in three brain regions: PFC, DS, and VS after long-term HAL treatment. **k** SMS activity in three brain regions: PFC, DS, and VS after long-term HAL treatment (*p < 0.05, ^#^p < 0.01, ^$^p < 0.001; n = 8–10 animals/group).

A major problem of current APD therapy of schizophrenia is the frequently observed failure in the clinic, for which mechanisms have only recently started to emerge [30]. Here we tested for a potential sphingolipid involvement. In a model of APD treatment failure (Fig. 4a), long-term treatment with HAL for 14 days abrogated its therapeutic effects, expressing an increase in locomotion boost not only for AMPH-VEH, but also for AMPH-HAL-treated rats. The long-term treatment abrogated HAL therapeutic effects as no inhibition of the AIH was observed any more (ANOVA, Factor *Group*, F_2,48_ = 8.292, p < 0.001, Factor *Time*, F_1,48_ = 0.903, p = 0.347, Interaction, F_4,72_ = 2.566, p = 0.088, Fig. 4b). Over the last 20 min of AIH testing, the HAL-treated group showed a noticeable drop in locomotor activity (p = 0.002, p < 0.001, p < 0.001 for 30, 35 and 40 min, respectively, vs controls, Fig. 4b). However, pre-planned comparisons of % AUC values, as groups differed in locomotion at the end of the baseline, revealed a significant increase in locomotion boost for AMPH-HAL (p < 0.001) and AMPH-VEH (p = 0.044) vs SAL-VEH in the first 20 min of AMPH challenge in AIH test, but not in the second 20 min (p > 0.05; Figs. 4c and S3).

AMPH sensitization did not cause altered emotional behavior in the AIH when tested after 14 days. Evaluation of center time spending in OF arena in AIH showed the higher anxiety level of rats treated with HAL 20-40 min after the AMPH challenge, as indicated by an ANOVA (factors: *Time*, F_2,72_ = 29.342, p < 0.001, *Group*, F_2,72_ = 6.229, p = 0.003, *Interaction*, F_4,72_ = 0.648, p = 0.630; Fig. 3d) and AUC analysis (p = 0.012 AMPH-HAL vs. SAL-VEH, Fig. 4e).

AMPH sensitization did not affect PPI significantly after 14 days (Fig. 4f). The AUC analysis of PPI nevertheless revealed a dramatic rise after long-term HAL treatment compared to the control group, although no sensorimotor deficit was observed in untreated rats (P100 dB, pp 74 dB: p < 0.001, AMPH-HAL vs. SAL-VEH, Fig. 4f). An ANOVA demonstrated a significant effects of factor *ppSt* (F_2,72_ = 26.193, p < 0.001) and factor *Group* (F_2,72_ = 8.388, p < 0.001), but not for the Interaction (F_4,72_ = 1.548, p = 0.198).

### No role of sphingolipids in APD failure

**Fig. 5:**
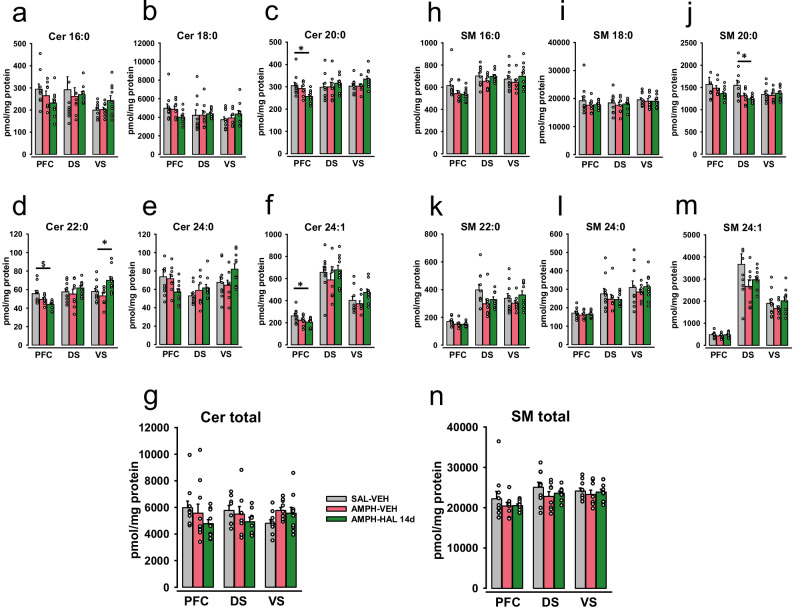
The concentrations of ceramide and sphingomyelin species after long-term chronic (14 days) haloperidol treatment in three brain structures: PFC, DS and VS. Data are presented as means ± SEM. **a** Levels of ceramide 16:0 (Cer 16:0). **b** Levels of ceramide 18:0 (Cer 18:0). **c** Levels of ceramide 20:0 (Cer 20:0). **d** Levels of ceramide 22:0 (Cer 22:0). **e** Levels of ceramide 24:0 (Cer 24:0). **f** Levels of ceramide 24:1 (Cer 24:1). **g** Levels of total ceramide (Cer Total). **h** Levels of sphingomyelin 16:0 (SM 16:0). **i** Levels of sphingomyelin 18:0 (SM 18:0). **j** Levels of sphingomyelin 20:0 (SM 20:0). **k** Levels of sphingomyelin 22:0 (SM 22:0). **l** Levels of sphingomyelin 24:0 (SM 24:0). **m** Levels of sphingomyelin 24:1 (SM 24:1). **n** Levels of total sphingomyelin (SM Total). Data were analyzed by one-way ANOVA followed by LSD pre-planned comparisons with Bonferroni’s correction (n = 8-10 animals/group; *p < 0.05, ^#^p < 0.01, ^$^p < 0.001).

No significant elevation of the sphingolipid enzyme activities was found 14 days after psychosis induction (p > 0.05; Fig. 4g–k), where only a decrease in AC activity in the VS was observed (F_2,25_ = 4.518, p = 0.021; AMPH-VEH vs SAL-VEH: p = 0.009, Fig. 4i). When psychosis-induction and APD treatment effects were no longer visible, sphingolipid responses in the brain were also largely diminished (Fig. 5). The AMPH-sensitization alone had no significant effects on Cer or SM levels in the PFC, VS, and DS (p > 0.05). Only after long-term HAL treatment, effects emerged. Cer 20:0 (p = 0.009), Cer 22:0 (p = 0.002), and Cer 24:1 (p = 0.022) levels were significantly decreased in the PFC (Fig. 5c–f). Remarkably, the Cer 22:0 concentration was raised in the VS in the same group of animals (p = 0.023, Fig. 5d). We further report decreased levels of SM 20:0 in the DS after AMPH-HAL long-term treatment (p = 0.009; Figs. 5j and S4). Altogether, sphingolipid changes coincide with the observation of psychotic-like behavioral symptoms, but vanish when induced symptoms are no longer present.

### ASM inhibition with KARI201 reverses psychosis-like behavior

**Fig. 6:**
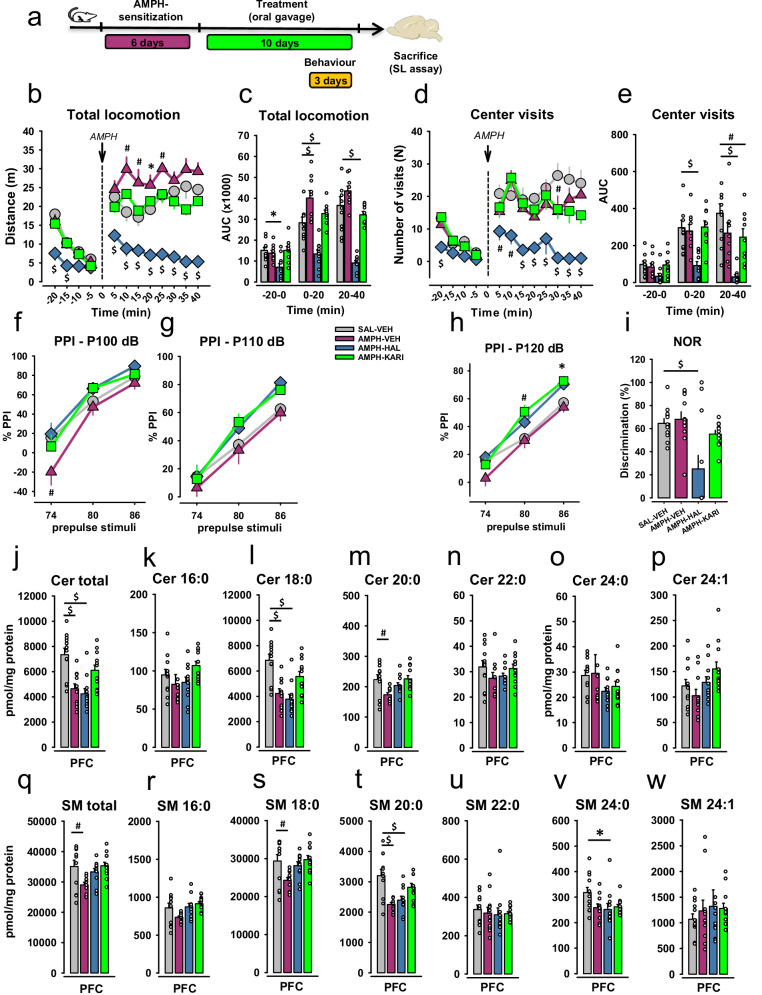
The selective ASM inhibitor KARI201 has antipsychotic effects in a rat model of schizophrenia. Data are presented as means ± SEM. **a** Study design for the experiment with HAL and KARI201 treatment. **b**. Total locomotion of animals in AMPH-induced hyperlocomotion (AIH) test with 10-day treatment (oral gavage, per os). One group was a control (SAL-VEH), and three groups were sensitized with AMPH and treated with either VEH (AMPH-VEH), HAL at 1 mg/kg (AMPH-HAL), or KARI201 at 10 mg/kg (AMPH-KARI). **c** The area under the curve (AUC) for total locomotion represents the baseline level (Bl), first and second 20 min after the AMPH challenge. Two-way ANOVA of the AUC analysis of locomotion indicated significant effects by factors *Time* (F_2,117_ = 65.427, p < 0.001), *Group* (F_3,117_ = 50.167, p < 0.001), and *Time* *×* *Group* interaction (F_6,117_ = 6.778, p < 0.001). The AMPH-VEH group was more active during the first 20 min after AMPH injection compared to SAL-VEH rats (^#^p < 0.001), while HAL-treated rats demonstrated a dramatic drop in AUC locomotion in comparison with the control group (p < 0.001). **d** Visits made in the central zone of the open field (Center visits) in the AIH test after VEH or HAL or KARI treatment. **e** AUC for visits in the central zone of the open field (Center visits) in the AIH test. AUC analysis revealed a significant effect of factors *Time* (F_2,117_ = 28.716, p < 0.001), *Group* (F_3,117_ = 21.858, p < 0.001), and *Time* *×* *Group* interaction (F_6,117_ = 3.465, p = 0.004). The AMPH-HAL group demonstrated lower number of center visits during AMPH challenge period (p < 0.001), while the AMPH-KARI group showed the same drop but only at the second 20-min interval of AMPH challenge period (p < 0.008) vs. controls. **f**–**h** Prepulse inhibition (PPI) of acoustic startle for three pulse stimuli: 100, 110, and 120 dB, and for three prepulse stimuli: 74, 80, and 86 dB. The lower the percentage of PPI the higher the sensorimotor gating animals demonstrate. **i** The discrimination level between novel and familiar objects in the novel object recognition (NOR) test to evaluate short-term memory. **j**–**p** Levels of ceramide species (Cer total, Cer 16:0, Cer 18:0, Cer 20:0, Cer 22:0, Cer 24:0, Cer 24:1) in AMPH-sensitized animals treated with VEH (AMPH-VEH) or HAL (AMPH-HAL) or KARI201 (AMPH-KARI) and controls (SAL-VEH). Brain samples were collected from the prefrontal cortex (PFC). **q**–**w** Levels of sphingomyelin species (SM total, SM 16:0, SM 18:0, SM 20:0, SM 22:0, SM 24:0, SM 24:1) in AMPH-sensitized animals treated with VEH (AMPH-VEH) or HAL (AMPH-HAL) or KARI201 (AMPH-KARI) and controls (SAL-VEH). Brain samples were collected from the prefrontal cortex (PFC). Data were analyzed by one- or two-way ANOVA followed by LSD pre-planned comparisons with Bonferroni’s correction. (n = 10-12 animals/group; *p < 0.05, ^#^p < 0.01, ^$^p < 0.001).

To further investigate the relationship between ASM activity and psychotic symptoms, we used the novel selective ASM inhibitor, KARI201 [33] and compared it with the effects of HAL treatment. Inducing an AMPH-psychosis in a rat model significantly enhanced AIH responses in the rats. This effect could be blocked by short-term chronic treatment with HAL. However, HAL reduced locomotor activity already at baseline level. KARI201 (10 mg/kg) [33] short-term treatment also reduced AIH responses to the level of vehicle controls. Interestingly, KARI201 did this without affecting locomotor baseline levels (Fig. 6b, c). An ANOVA of locomotor activity showed a significant effect of factors *Time* (F_3,156_ = 40.662, p < 0.001) and *Group* (F_3,156_ = 18.060, p < 0.001), but not *Time×Group* interaction (p > 0.05), during the baseline period. There was a significantly lower level of locomotion in the AMPH-HAL group compared to control rats during baseline after 5 min (p < 0.001) and 10 min (p < 0.001) of testing, which however, normalized thereafter (p > 0.05; Fig. 6b). No significant difference was observed between the AMPH-KARI201 and SAL-VEH groups compared to controls at the baseline (p > 0.05). The AMPH challenge induced a significant increase in locomotion (ANOVA, factor *Group*: F_3, 312_ = 152.541, p < 0.001). This was most pronounced in the AMPH-VEH group (10, 15, 20, and 25 min after the AMPH injection: p < 0.001, p = 0.002, p = 0.012, and p = 0.008 vs. SAL-VEH). The increase was significantly attenuated in the AMPH-HAL group (all time points after the AMPH injection: p < 0.001 at vs. SAL-VEH). No difference was observed between KARI201-AMPH and the SAL-VEH groups (p > 0.05). These results were confirmed in the AUC analysis (Figs. 6c and S5).

HAL treated animals showed an increase in anxiety levels at baseline as indicated by reduced number of center visits. An ANOVA indicated a significant effect of factors *Time* (F_3,156_ = 26.340, p < 0.001) and *Group* (F_3,156_ = 8.796, p < 0.001), but not *Time×Group* interaction (p > 0.05), during the baseline period. The AMPH-HAL group made less center visits at first 5 min of testing (p < 0.001 vs. SAL-VEH). The AIH challenge increased center visits. This was not affected by KARI201, but significantly reduced by HAL treatment (Fig. 6d, e). After AMPH challenge, there was a significant effect of factor *Group* (F_3,312_ = 51.609, p < 0.001), but not *Time* or *Time×Group* interaction (p > 0.05). The AMPH-HAL group differed from SAL-VEH 5 min (p = 0.007), 10 min (p = 0.004), and 15-40 min (p < 0.001) after AMPH injection. We suggest that effect of HAL is explained not by higher anxiety levels of animals but by a prominent sedative action of the drug, which was also observed in general locomotor activity.

In the same animals, the sensorimotor gating deficit, induced by the AMPH sensitization, was rescued by both, HAL and KARI201 (Fig. 6f–h). The PPI for the pp+P pair 74 + 100 dB was significantly decreased in the AMPH-VEH group (p = 0.002 vs SAL-VEH, Fig. 6f). This was reversed by both HAL and KARI201 treatment (p > 0.05 vs. SAL-VEH). No significant differences between the groups in PPI levels was observed for 110 dB pulse intensity (Fig. 6g). While the AMPH sensitization did not yield a significant decline in PPI at 120 dB pulse stimulus, HAL and KARI201 treatment significantly enhanced the PPI at a 80 and 86 pre-pulse stimulus intensity (p = 0.003 and p = 0.015 vs. SAL-VEH; Fig. 6h). These findings suggest that KARI201 has a similar antipsychotic potential as HAL.

While the AMPH sensitization did not affect cognitive function in the NOR test, treatment with HAL caused a significant decline (p < 0.001 vs. SAL-VEH; Fig. 6i). This was not seen in the KARI201-treated animals. This may suggest that KARI201, at a therapeutically active dose, may have less cognitive impairing side effects than HAL [55].

### Brain sphingolipids after KARI201 treatment

Given that the key alterations in ASM activity and sphingolipid levels were PFC-specific, we measured the effects of an ASM inhibitor, KARI201, on ceramide and SM levels in the PFC compared to HAL treatment. Similar to the results of our first experiment, a notable reduction in both ceramide and SM levels were revealed for the AMPH-sensitized VEH treated group (AMPH-VEH) that demonstrated psychotic-like symptoms in the behavioral tests. Total ceramides were decreased in AMPH-VEH (p < 0.001) and AMPH-HAL (p < 0.001) groups in comparison with controls (SAL-VEH), showing the significant effect of factor *Group* (F_3;42_ = 10.779, p < 0.001) and suggesting no impact of HAL treatment (Fig. 6j). The most abundant Cer 18:0 species displayed the same aberrations (*Group*: F_3;42_ = 11.334, p < 0.001) in AMPH-VEH (p < 0.001) and AMPH-HAL (p < 0.001) animals (Fig. 6l). Cer 20:0 levels were diminished only in AMPH-VEH rats (p = 0.004), significantly affected by factor *Group* (F_3;42_ = 4.410, p = 0.009) (Fig. 6m). Other ceramide species were impacted neither by AMPH-sensitization nor by treatment (Figs. 6k, n–p and S6).

Levels of total SM and few SM species were found to be remarkably decreased in psychotic-like animals (Fig. 6q–w). An ANOVA revealed a significant effect of factor *Group* for total SM (F_3;42_ = 4.205, p = 0.011) and SM 18:0 (F_3;42_ = 4.149, p = 0.012) with a strong decline in its levels for AMPH-VEH group (p = 0.004 and p = 0.005, respectively) (Fig. 6q, s). We also observed a similar drop in SM 20:0 levels in both AMPH-VEH (p < 0.001) and AMPH-HAL (p < 0.001) animals (*Group*: F_3;42_ = 9.101, p < 0.001) (Fig. 6t). Interestingly, that SM 24:0 concentrations were diminished only in AMPH-HAL group compared to SAL-VEH (p = 0.011) (Fig. 6v). We reported no alterations in the levels of SM 16:0, SM 22:0, and SM 24:1.

Altogether, we identified the striking effect of AMPH-sensitization on the sphingolipid balance in the PFC, similarly to the previous findings. The majority of alterations were independent of HAL treatment. However, ASM inhibition through KARI201 administration recovered all ceramides and SMs to the levels of control animals.

### Gene expression in the PFC after AMPH-induced psychosis and APD treatment

**Fig. 7:**
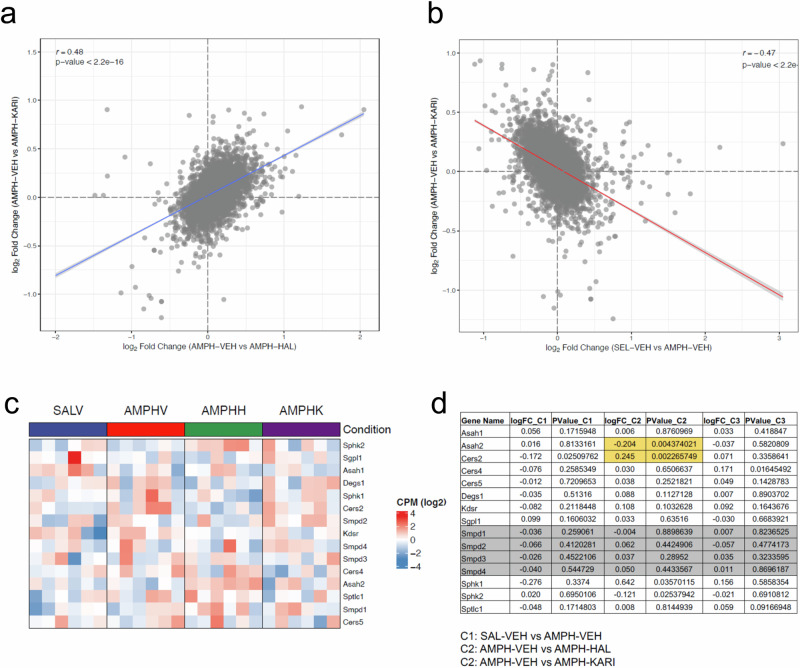
Amphetamine (AMPH)-induced psychosis alters gene expression in the prefrontal cortex of rats. Antipsychotic (APD) treatment with haloperidol (HAL/H) or KARI201 (KARI/K) does not reverse these effects, but has its own expression profile. Animals were tested at a time of confirmed psychotic-like state and efficacy of the APD treatment (n = 6/group). **a**, **b** Differential expression analysis of RNA sequencing data for all genes analyzed. **c**, **d** Selective analysis of sphingolipid controlling genes.

Membrane sphingolipids regulate classical transmitter signaling through lipid membranes of the brain [17, 56]. As the observed changes in sphingolipid regulation cannot fully explain behavioral alterations after psychosis-like state induction and its reversal with HAL or KARI201, we measured gene expression in the PFC using RNA sequencing [57], after psychosis induction and successful APD treatment. Despite a considerable interindividual variance, results showed that psychosis-induction was accompanied by a significant upregulation of the genes Slc2a5, Pld4, Olig1, Fgfr1, Gpr17, Cxcl14, Phlda3, Gna12, Abca2, Sox1, RGD1566085 and a down-regulation of Dpm2, Ergic2, Rab2a, Vma21 when AMPH-VEH was compared to the SAL-VEH group (Fig. 7a, b). The HAL treatment did not reverse any of these effects, but upregulated expression of Col6a3, Slc22a8, Ndufs5-ps2, Net1, Rasl11b, and downregulated Leo1 and Bmal1 expression in the comparison of the AMPH-HAL vs. AMPH-VEH groups. KARI201 treatment did also not reverse the effects of psychosis-induction. Nor did it share effects with the HAL treatment. Instead, the KARI201 treatment upregulated expression of Tmem238 and Nr2f6 in the comparison of the AMPH-KARI vs. AMPH-VEH groups. These data may suggest that psychosis induction is accompanied by numerous changes in gene expression on the PFC. Successful APD treatment does not work by a reversal of these changes, but by other mechanisms. Despite a shared effect on ASM activity in the PFC, the new ASM targeting antipsychotic drug KARI201 does not share effects on gene expression with HAL.

A selective analysis of sphingolipid controlling genes did not show different expression after psychosis induction (AMPH-VEH vs. SAL-VEH). However, it showed a selective reduction in the expression of Asah2 and an enhanced expression of Cers2 after HAL treatment (AMPH-HAL vs. AMPH-VEH). KARI201 did not affect sphingolipid controlling genes (AMPH-KARI vs. AMPH-VEH). Interestingly, none of the treatments affected the expression of the ASM coding gene Smpd1 (Figs. 7c, d and S7).

## Discussion

Schizophrenia is a highly heterogeneous psychiatric disorder, which is elicited by multiple genetic and non-genetic factors. It remains insufficiently treatable with the current pharmaceutical approaches. In this study, we describe a new pathological mechanism and identify a new pharmacological treatment target with the use of a recently introduced ligand for it. An initial analysis of human gene polymorphisms and brain gene expression in schizophrenia patients identified an association of *SMPD1* and *SMPD3* genes coding for ASM and NSM. In a rat model of amphetamine-induced schizophrenia, we found a locally restricted increase of ASM activity in the PFC that responds to psychosis-induction and its treatment. Chronic HAL treatment reversed behavioral symptoms and the ASM activity increase. A sphingolipidomic analysis confirmed a disrupted ceramide metabolism in the PFC during psychosis. Targeting enhanced ASM activity in a psychotic-like state with the selective ASM inhibitor KARI201 reversed psychotic-like behavior. While effective HAL treatment led also to a locomotor decline and cognitive impairments, KARI201 did not.

Numerous genes coding for proteins in rather distinct pathways of the brain have been associated with schizophrenia and the APD treatment response [58–60]. However, little attention was given to genes related to lipid regulatory pathways. Based on recently shown involvement of the sphingolipid controlling enzymes that tightly regulate sphingolipids in brain cellular membranes in symptom dimensions of schizophrenia [51, 61], we investigated those genes for an association with schizophrenia diagnosis and the APD treatment response. Our results showed a selective association of *SMPD3* polymorphisms with a schizophrenia diagnosis and of *SMPD1* expression in the PFC. In human studies, the downregulation of seven genes associated with glycosphingolipids/sphingolipids has been reported in schizophrenia [24]. Surprisingly, the products of some of these genes, such as *SGPP1*, *GALC* and *UGT8*, have opposite effects on ceramide synthesis. However, all the patients investigated were under APD treatment. Thus, the observations might be best explained by the therapeutic action of the APD drugs, regulating not only the DA system, but also sphingolipid metabolism.

Although we found an attenuated expression of SMPD1 in the PFC of schizophrenic patients, it might not correspond directly with the activity level of ASM in the same brain region. Thus, we showed previously that despite an enhanced ASM activity in the PFC of APP/PS1 mice, the mRNA levels of the SMPD1 gene were not altered in comparison with wild type animals [33]. We, therefore, suggest that different mechanisms might be involved in the regulation of gene expression and enzyme activity. Given the chronic APD treatment of schizophrenic patients, we hypothesize a considerable impact of the medications on the study outcomes, which is of interest for future investigation. Moreover, evidence suggests that the duration of the disorder influences substantially the reduction in expression levels of several genes coding for sphingolipid enzymes. The authors speculate that on the later stages of the disease progression some compensatory mechanisms might contribute to the elimination of abnormal gene expression [24].

To further characterize the nature of sphingolipid involvement in psychosis-induction and APD-treatment, we used animal models of psychosis combined with APD treatment efficacy and failure [30, 31, 54]. In this model, an AMPH-induced psychosis was characterized by potentiated AIH response and a PPI deficit up to 7 days after induction. At that time, we observed an increase of ASM activity in the PFC, of AC activity in the VS and SMS activity in the VS. These responses were no longer observed 14 days after AMPH sensitization, when also behavioral symptoms had waned.

In this study, we utilized a pharmacological animal model of schizophrenia, where psychotic-like symptoms were induced after a 6-day AMPH sensitization procedure. We found that the most significant effects on behavior and sphingolipid metabolism were observed after one week of AMPH withdrawal. We suggest, that the diminishing differences in enzyme activity and Cer- and SM levels in a 14-day period may be attributed to the gradual recovery of the animals from the AMPH-induced deterioration. Employing of genetic or developmental animal models of schizophrenia might shed light on the long-term effects of the disorder’s pathogenesis and the APD treatment.

The temporal association observed in our research may give a first clue of regional ASM involvement in psychosis-induction. Enhanced ASM activity, however, was observed not only in schizophrenia, but also in other psychiatric and neurodegenerative disorders, such as depression [18, 48], addiction [56], and Alzheimer’s disease [33, 62]. Experiments with ASM over-expressing mice showed diminished neurogenesis, neuronal maturation, and survival that were accompanied by depression-like behavior [18, 63]. The functional focus of this ASM hyperactivity and ceramide dysregulation was identified in the hippocampus and amygdala [18, 64]. Although, we have not investigated the ASM activity in the hippocampus in our model of AMPH-induced psychosis, it is of great interest for the future research. Hippocampal hyperactivity might play a crucial role in the pathogenesis of psychotic disorders, including schizophrenia, through the upregulation of dopaminergic activity and disruption of neuronal circuits [65]. Previously, we showed elevated dopamine levels in the hippocampus in the same rat model [28]. Moreover, enhanced ASM activity in this region was observed in animal models of major depression and of Alzheimer’s disease [18, 33].

Given the multifaced nature of schizophrenia, manifesting through a great variety of symptoms, we suggest that the effects of the ASM hyperactivity on other abnormal behaviors, such as stereotypic and repetitive behavior, catatonia and catalepsy or social withdrawal, should be investigated in future studies. For instance, we previously demonstrated a sex-specific impact of ASM over-expression on social behavior in mice. Female, but not male tgASM mice, exhibited a deficit in social preference, accompanied by anxiety- and depression-like behavior. These abnormalities were successfully restored by the functional ASM inhibitor amitriptyline [66]. The impaired social behavior and sex-specific differences and impact of the direct ASM inhibition on it need to be further explored in animal models of psychosis.

In the present study, sphingolipidomic analysis showed a time-dependent pattern of ceramide changes with a strong focus on the PFC, rather than DS or VS. In the PFC, AMPH sensitization induced a decline in several ceramide species and in total ceramide levels. This was no longer observed when psychosis-like behavior was gone. Little changes were observed for the more abundant SM species. These observations may further localize the PFC as a key spot for ASM and ceramide dysfunction in schizophrenia induction, which may support previous localization studies [67, 68]. The current findings would directly expand the membrane theory of schizophrenia, which in its original form claims that a dysfunction in biological membranes of the brain and periphery, caused by phospholipid pathology, is the initial event of schizophrenia pathogenesis [69]. Here we extend this view by another class of membrane lipids that, together with phospholipids and cholesterol, dynamically shape the membrane micro-environment of basically all signaling-related proteins in synaptic membranes of the brain [16, 17].

While prior studies noted increased ceramide in the frontal cortex of schizophrenia patients, it was observed exclusively in white matter [21], although recent findings suggest lower Cer in gray matter, consistent with our results [70]. Despite increased ASM activity and reduced Cer levels in the PFC during psychotic behaviors, our results align with studies on forebrain specific ASM over-expressing mice showing higher ASM activity, but minimal Cer change. Alterations in sphingolipid metabolizing enzymes, such as reduced NSM and glucosyl-ceramidase as well as increased sphingosine-1-phosphate lyase, may explain the Cer decreases [71].

During treatment efficacy, i.e. after 7-day treatment, HAL effectively reversed psychosis-like behavior. Among sphingolipid changes induced by AMPH sensitization, only the PFC ASM activity increase was effectively remitted. In addition, HAL decreased NSM activity in the PFC, and increased SMS activity in the DS. During treatment failure, i.e. after 14-day treatment, HAL had no effects on enzyme activity any more. Many effects on ceramide levels, however, were preserved at this time. This may suggest that HAL treatment works rather directly on ASM activity, but only with some delay on bioactive ceramide levels. Altered ceramide and subsequent changes in neuronal membrane function [17], may also be more relevant for schizophrenia induction, than for its reversal after APD treatment. In general, HAL effects are consistent with the previous reports of functional inhibitory activity of ASM, found for many available APDs [72]. Such substances are characterized by specific physicochemical properties, which allow them to accumulate in lysosomes of the brain [32, 73]. After accumulation, they physically detach ASM from the inner leaf of the lysosomal membrane, leading to its deactivation [74]. Thus, we suggest that ASM inhibition in the PFC might, in addition to the DA D2 antagonism [75], be a parallel mode of action for many APDs mediating their full therapeutic activity.

Clinical Antipsychotic Trials of Intervention Effectiveness (CATIE) studies reported that a range of 15-60% of patients discontinue APD treatment due to its inefficacy within 18 months [76, 77]. Animal studies with HAL and olanzapine demonstrated restoration of the enhanced locomotor activity and sensorimotor gating after short-term chronic treatment, while the drugs lose their effects after long-term chronic administration [30, 31, 54]. Authors suggested that extracellular striatal DA concentrations correspond to APD effects more precisely than DA D2 receptor occupancy. Moreover, it was found that the efficacy of the drugs was associated with inhibition of the DA transporter (DAT) and synaptic vesicle release, while the APD failure was linked to the opposite outcomes. Administration of a DAT inhibitor combined with HAL for 14 days recovered the efficacy of the APD treatment [10, 30]. However, also a role of serotonin (5-HT) adaptations has been discussed for APD efficacy [78, 79]. The ASM may link the sphingolipid system with monoaminergic transmission in schizophrenia treatment. A close ASM control of DA and 5-HT signaling has been shown in the brain [17, 56, 80].

In order to directly test the role of ASM in APD treatment, we used the recently developed selective ASM inhibitor KARI201 [33]. Short-term chronic treatment with KARI201 was equally effective in reducing psychosis-like behavior as HAL. This may further confirm enhanced ASM activity as a treatment target for schizophrenia. Effective HAL treatment, however, can be accompanied by locomotor sedation, anxiety, and cognitive deficits [81]. None of these side effects were observed with KARI201 in an effective therapeutic dose. Moreover, in our previous research, KARI201 showed a significant improvement in learning and memory in the Morris water maze and fear-conditioning test, where the APP/PS1 mice with cognitive impairments were assessed. The observed effects have been associated with the improvement in hippocampal neurogenesis and synapse plasticity throughout the stimulation of ghrelin receptors by KARI201 [33]. These findings suggest potential pro-cognitive effects of ASM inhibition and require further investigation of it using other animal models of schizophrenia with pronounced cognitive-like symptoms.

In addition, we demonstrated previously no direct impact of this substance on locomotor function of the mice tested in the Morris water maze after 1,5 months of treatment, indicating the low probability of adverse effects associated with a dysfunctional extrapyramidal system. We also found very low affinity to dopamine receptors (DRD1, DRD2L, DRD4.4, DRD5), suggesting neglectable effects on motor functions [33].

Furthermore, KARI201 effectively reversed the sphingolipid profile associated with an induced schizophrenia-like state. This may qualify the ASM inhibitor KARI201 as a potential new treatment for schizophrenia. Due to the absence of activity in the dopamine system, KARI201 might mediate its antipsychotic action through the recovery of membrane properties of PFC neurons which, in turn, regulate activity of dopaminergic neurons in the mesolimbic system. Although the pharmacokinetics and safety profile has been already characterized in vivo, the precise mode of action for KARI201 has to be meticulously explored in a series of preclinical models before considering clinical trials.

In order to identify gene expression and protein mechanisms of psychotic states and its treatment, we performed an RNA Seq analysis in the PFC of rats. The results suggest that the AMPH-induced psychotic-like state was paralleled by a dysregulation of 15 genes. About half of them has been previously associated with schizophrenia in humans, such as Olig1 [82, 83], Fgfr1 [84–86], Gpr17 [87, 88], Gna12 [87], Abca2 [89], Sox1 [90], Dpm2 [91], and, as a locus for de novo mutations, Rab2a [92]. This may support the translational value of the AMPH-induced psychosis paradigm as an animal model of schizophrenia. Beyond that, the model adds numerous other genes as potential candidates for schizophrenia. Interestingly, no sphingolipid regulating gene emerged in a focused analysis. This might suggest that the discrepancy between ASM hyperactivity and Cer levels in the PFC may not easily be explained by compensatory regulations in the expression of genes of the sphingolipid rheostat.

None of the genes that was differently expressed after AMPH-induced psychosis was remitted in its expression by the HAL treatment. However, HAL action dysregulated other genes also associated with schizophrenia in humans, such as Col6a3 [93], Slc22a8 [94, 95], and Bmal1 [96, 97]. Although KARI201 did not share gene expression effects with HAL, it affected Nr2f6a, a previously identified schizophrenia gene [98]. This may support the view that HAL and KARI201 exert their antipsychotic effects on distinct pathways of the multi-focal schizophrenia pathology with little overlap and no direct involvement of sphingolipid regulating gene expression.

There are several limitations to this study. Although the AMPH-induced psychosis model is instrumental in examining specific effects associated with schizophrenia, it predominantly addresses positive symptoms and cognitive deficits related to attention, failing to encapsulate the full intricacy of the disorder and offering limited insights into its underlying etiology. Furthermore, in human post-mortem studies, enhanced levels of ceramide were demonstrated in the white matter of schizophrenic patients [21]. We found decreased levels of almost all ceramide species in the PFC gray matter, regardless of the treatment. However, in our study, we did not analyze subregions and cell types where ceramide was dysregulated. Thus, apart from the measured five enzymes that regulate conversation between ceramide and SM and sphingosine, the enzymes from the de novo sphingolipid synthesis and salvage pathways, such as ceramide synthase and glycosyltransferases, might also affect the final concentrations of ceramide by a stronger downregulation than upregulation [99].

## Conclusions

Taken together, we suggest a new mechanism for an AMPH-induced psychosis that is driven by a selective increase of ASM activity in the PFC and local dysfunction of numerous ceramide species. Behavioral effects and enzyme activation can be reversed by the APD treatment with HAL, however, with emerging side effects of locomotor sedation and cognitive deficits. Direct and selective ASM inhibition with the novel ligand KARI201 yields antipsychotic action, without common APD adverse effects. We, therefore, suggest that the newly explored mode of action via ASM inhibition should be considered as an alternative treatment for schizophrenia.

## Supplementary information

Supplemental material

## References

[1] World Health Organization. International Classification of Diseases, Eleventh Revision (ICD-11), Geneva; 2022.

[2] Jauhar S, Johnstone M, McKenna PJ. Schizophrenia. Lancet. 2022;399:473–86. 35093231 10.1016/S0140-6736(21)01730-X

[3] Owen MJ, Sawa A, Mortensen PB. Schizophrenia. Lancet. 2016;388:86–97. 26777917 10.1016/S0140-6736(15)01121-6 PMC4940219

[4] Howes OD, Onwordi EC. The synaptic hypothesis of schizophrenia version III: a master mechanism. Mol Psychiatry. 2023;28:1843–56. 37041418 10.1038/s41380-023-02043-w PMC10575788

[5] Creese I, Burt DR, Snyder SH. Dopamine receptor binding predicts clinical and pharmacological potencies of antischizophrenic drugs. Science. 1976;192:481–3. 3854 10.1126/science.3854

[6] Seeman P, Lee T, Chau-Wong M, Wong K. Antipsychotic drug doses and neuroleptic/dopamine receptors. Nature. 1976;261:717–9. 945467 10.1038/261717a0

[7] Lora A, Kohn R, Levav I, McBain R, Morris J, Saxena S. Service availability and utilization and treatment gap for schizophrenic disorders: a survey in 50 low- and middle-income countries. Bull World Health Org. 2012;90:47–54. 22271964 10.2471/BLT.11.089284 PMC3260570

[8] Loryan I, Melander E, Svensson M, Payan M, König F, Jansson B, et al. In-depth neuropharmacokinetic analysis of antipsychotics based on a novel approach to estimate unbound target-site concentration in CNS regions: link to spatial receptor occupancy. Mol Psychiatry. 2016;21:1527–36. 26809840 10.1038/mp.2015.229

[9] Samara MT, Nikolakopoulou A, Salanti G, Leucht S. How many patients with schizophrenia do not respond to antipsychotic drugs in the short term? An analysis based on individual patient data from randomized controlled trials. Schizophrenia Bull. 2019;45:639–46. 10.1093/schbul/sby095 PMC6483567 29982701

[10] Chestnykh D, Amato D, Kornhuber J, Müller CP. Pharmacotherapy of schizophrenia: mechanisms of antipsychotic accumulation, therapeutic action and failure. Behav Brain Res. 2021;403:113144. 33515642 10.1016/j.bbr.2021.113144

[11] Simpson EH, Kellendonk C, Kandel E. A possible role for the striatum in the pathogenesis of the cognitive symptoms of schizophrenia. Neuron. 2010;65:585–96. 20223196 10.1016/j.neuron.2010.02.014 PMC4929859

[12] Nestler EJ, Hyman SE. Animal models of neuropsychiatric disorders. Nat Neurosci. 2010;13:1161–9. 20877280 10.1038/nn.2647 PMC3750731

[13] Terry AV. Role of the central cholinergic system in the therapeutics of schizophrenia. Curr Neuropharmacol. 2008;6:286–92. 19506725 10.2174/157015908785777247 PMC2687934

[14] Davis KL, Stewart DG, Friedman JI, Buchsbaum M, Harvey PD, Hof PR, et al. White matter changes in schizophrenia: evidence for myelin-related dysfunction. Arch Gen Psychiatry. 2003;60:443–56. 12742865 10.1001/archpsyc.60.5.443

[15] Flynn SW, Lang DJ, Mackay AL, Goghari V, Vavasour IM, Whittall KP, et al. Abnormalities of myelination in schizophrenia detected in vivo with MRI, and post-mortem with analysis of oligodendrocyte proteins. Mol Psychiatry. 2003;8:811–20. 12931208 10.1038/sj.mp.4001337

[16] Kalinichenko LS, Abdel-Hafiz L, Wang AL, Mühle C, Rösel N, Schumacher F, et al. Neutral sphingomyelinase is an affective valence-dependent regulator of learning and memory. Cereb Cortex. 2021;31:1316–33. 33043975 10.1093/cercor/bhaa298

[17] Kalinichenko LS, Kornhuber J, Sinning S, Haase J, Müller CP. Serotonin signaling through lipid membranes. ASC Chem Neurosci. 2024;15:1298–320. 10.1021/acschemneuro.3c00823 PMC10995955 38499042

[18] Gulbins E, Palmada M, Reichel M, Lüth A, Böhmer C, Amato D, et al. Acid sphingomyelinase-ceramide system mediates effects of antidepressant drugs. Nature Med. 2013;19:934–38. 23770692 10.1038/nm.3214

[19] Zhuo C, Tian H, Chen J, Li Q, Yang L, Zhang Q, et al. Associations of cognitive impairment in patients with schizophrenia with genetic features and with schizophrenia-related structural and functional brain changes. Front Genetics. 2022;13:880027. 10.3389/fgene.2022.880027 PMC9437456 36061201

[20] Prabakaran S, Swatton JE, Ryan MM, Huffaker SJ, Huang JTJ, Griffin JL, et al. Mitochondrial dysfunction in schizophrenia: evidence for compromised brain metabolism and oxidative stress. Mol Psychiatry. 2004;9:684–97. 15098003 10.1038/sj.mp.4001511

[21] Schwarz E, Prabakaran S, Whitfield P, Major H, Leweke FM, Koethe D, et al. High throughput lipidomic profiling of schizophrenia and bipolar disorder brain tissue reveals alterations of free fatty acids, phosphatidylcholines, and ceramides. J Prot Res. 2008;7:4266–77. 10.1021/pr800188y 18778095

[22] Tessier C, Sweers K, Frajerman A, Bergaoui H, Ferreri F, Delva C, et al. Membrane lipidomics in schizophrenia patients: a correlational study with clinical and cognitive manifestations. Transl Psychiatry. 2016;6:e906. 27701405 10.1038/tp.2016.142 PMC5315538

[23] Schmitt A, Wilczek K, Blennow K, Maras A, Jatzko A, Petroianu G, et al. Altered thalamic membrane phospholipids in schizophrenia: a postmortem study. Biol Psychiatry. 2004;56:41–5. 15219471 10.1016/j.biopsych.2004.03.019

[24] Narayan S, Head SR, Gilmartin TJ, Dean B, Thomas EA. Evidence for disruption of sphingolipid metabolism in schizophrenia. J Neurosci Res. 2009;87:278–88. 18683247 10.1002/jnr.21822 PMC2606914

[25] Trubetskoy V, Pardiñas AF, Qi T, Panagiotaropoulou G, Awasthi S, Bigdeli TB, et al. Mapping genomic loci implicates genes and synaptic biology in schizophrenia. Nature. 2022;604:502–8. 35396580 10.1038/s41586-022-04434-5 PMC9392466

[26] Quinville BM, Deschenes NM, Ryckman AE, Walia JS. A comprehensive review: sphingolipid metabolism and implications of disruption in sphingolipid homeostasis. Int J Mol Sci. 2021;22:5793. 34071409 10.3390/ijms22115793 PMC8198874

[27] Li R, Ma X, Wang G, Yang J, Wang C. Why sex differences in schizophrenia? J Transl Neurosci. 2016;1:37–42. PMC5688947 29152382

[28] Uzuneser TC, Schindehütte M, Dere E, von Hörsten S, Kornhuber J, Grömer TW, et al. Schizophrenia dimension-specific antipsychotic drug action and failure in amphetamine-sensitized psychotic-like rats. Eur Neuropsychopharm. 2018;28:1382–93. 10.1016/j.euroneuro.2018.09.005 30243682

[29] Chestnykh D, Graßl F, Pfeifer C, Dülk J, Ebner C, Walters M, et al. Behavioural effects of APH199, a selective dopamine D4 receptor agonist, in animal models. Psychopharmacology. 2023;240:1011–31. 36854793 10.1007/s00213-023-06347-1 PMC10006056

[30] Amato D, Canneva F, Cumming P, Maschauer S, Groos D, Dahlmanns JK, et al. A dopaminergic mechanism of antipsychotic drug efficacy, failure, and failure reversal: the role of the dopamine transporter. Mol Psychiatry. 2020;25:2101–18. 30038229 10.1038/s41380-018-0114-5 PMC7473845

[31] Samaha AN, Seeman P, Stewart J, Rajabi H, Kapur S. “Breakthrough” dopamine supersensitivity during ongoing antipsychotic treatment leads to treatment failure over time. J Neurosci. 2007;27:2979–86. 17360921 10.1523/JNEUROSCI.5416-06.2007 PMC6672560

[32] Uzuneser TC, Weiss EM, Dahlmanns J, Kalinichenko LS, Amato D, Kornhuber J, et al. Presynaptic vesicular accumulation is required for antipsychotic efficacy in psychotic-like rats. J Psychopharmacol. 2021;35:65–77. 33274688 10.1177/0269881120965908 PMC7770212

[33] Park MH, Park KH, Choi BJ, Han WH, Yoon HJ, Jung HJ, et al. Discovery of a dual-action small molecule that improves neuropathological features of Alzheimer’s disease mice. Proc Nat Acad Sci USA. 2022;119:e2115082119. 35027452 10.1073/pnas.2115082119 PMC8784098

[34] Thiel CM, Müller CP, Huston JP, Schwarting RK. High vs. low reactivity to a novel environment: behavioural, pharmacological and neurochemical assessments. Neuroscience. 1999;93:243–51. 10430488 10.1016/s0306-4522(99)00158-x

[35] Simon P, Dupuis R, Costentin J. Thigmotaxis as an index of anxiety in mice. Influence of dopaminergic transmissions. Behav Brain Res. 1994;61:59–64. 7913324 10.1016/0166-4328(94)90008-6

[36] Kraeuter AK, Guest PC, Sarnyai Z. The open field test for measuring locomotor activity and anxiety-like behavior. Methods Mol Biol. 2019;1916:99–103. 30535687 10.1007/978-1-4939-8994-2_9

[37] Ang MJ, Lee S, Kim JC, Kim SH, Moon C. Behavioral tasks evaluating schizophrenia-like symptoms in animal models: a recent update. Curr Neuropharmacol. 2021;19:641–64. 32798374 10.2174/1570159X18666200814175114 PMC8573744

[38] Chen CH, Lee PW, Liao HM, Chang PK. Neuroligin 2 R215H mutant mice manifest anxiety, increased prepulse inhibition, and impaired spatial learning and memory. Front Psychiatry. 2017;8:257. 29230184 10.3389/fpsyt.2017.00257 PMC5711828

[39] Fu K, Miyamoto Y, Sumi K, Saika E, Muramatsu SI, Uno K, et al. Overexpression of transmembrane protein 168 in the mouse nucleus accumbens induces anxiety and sensorimotor gating deficit. PLoS ONE. 2017;12:e0189006. 29211814 10.1371/journal.pone.0189006 PMC5718521

[40] van der Werf IM, Van Dam D, Missault S, Yalcin B, De Deyn PP, Vandeweyer G, et al. Behavioural characterization of AnkyrinG deficient mice, a model for ANK3 related disorders. Behav Brain Res. 2017;328:218–26. 28411148 10.1016/j.bbr.2017.04.014

[41] Winters BD, Forwood SE, Cowell RA, Saksida LM, Bussey TJ. Double dissociation between the effects of peri-postrhinal cortex and hippocampal lesions on tests of object recognition and spatial memory: heterogeneity of function within the temporal lobe. J Neurosci. 2004;24:5901–8. 15229237 10.1523/JNEUROSCI.1346-04.2004 PMC6729235

[42] Dere E, Huston JP, De Souza Silva MA. The pharmacology, neuroanatomy and neurogenetics of one-trial object recognition in rodents. Neurosci Biobehav Rev. 2007;31:673–704. 17368764 10.1016/j.neubiorev.2007.01.005

[43] Braff D, Stone C, Callaway E, Geyer M, Glick I, Bali L. Prestimulus effects on human startle reflex in normals and schizophrenics. Psychophysiology. 1978;15:339–43. 693742 10.1111/j.1469-8986.1978.tb01390.x

[44] Swerdlow NR, Geyer MA. Using an animal model of deficient sensorimotor gating to study the pathophysiology and new treatments of schizophrenia. Schizophrenia Bull. 1998;24:285–301. 10.1093/oxfordjournals.schbul.a033326 9613626

[45] Fendt M, Fanselow MS. The neuroanatomical and neurochemical basis of conditioned fear. Neurosci Biobehav Rev. 1999;23:743–60. 10392663 10.1016/s0149-7634(99)00016-0

[46] Peleg-Raibstein D, Sydekum E, Russig H, Feldon J. Withdrawal from repeated amphetamine administration leads to disruption of prepulse inhibition but not to disruption of latent inhibition. J Neural Transm. 2006;113:1323–36. 16362632 10.1007/s00702-005-0390-5

[47] Mühle C, Kornhuber J. Assay to measure sphingomyelinase and ceramidase activities efficiently and safely. J Chromatography A. 2017;1481:137–44. 10.1016/j.chroma.2016.12.033 28012590

[48] Gulbins A, Schumacher F, Becker KA, Wilker B, Soddemann M, Boldrin F, et al. Antidepressants act by inducing autophagy controlled by sphingomyelin–ceramide. Mol Psychiatry. 2018;23:2324–46. 30038230 10.1038/s41380-018-0090-9 PMC6294742

[49] Schumacher F, Carpinteiro A, Edwards MJ, Wilson GC, Keitsch S, Soddemann M, et al. Stress induces major depressive disorder by a neutral sphingomyelinase 2-mediated accumulation of ceramide enriched exosomes in the blood plasma. J Mol Med. 2022;100:1493–508. 36045177 10.1007/s00109-022-02250-y PMC9470690

[50] Huston JP, Kornhuber J, Mühle C, Japtok L, Komorowski M, Mattern C, et al. A sphingolipid mechanism for behavioral extinction. J Neurochem. 2016;137:589–603. 26788861 10.1111/jnc.13537

[51] Kalinichenko LS, Gulbins E, Kornhuber J, Müller CP. Sphingolipid control of cognitive functions in health and disease. Prog Lipid Res. 2022;86:101162. 35318099 10.1016/j.plipres.2022.101162

[52] Lanz TA, Reinhart V, Sheehan MJ, Sukoff Rizzo SJ, Bove SE, James LC, et al. Postmortem transcriptional profiling reveals widespread increase in inflammation in schizophrenia: a comparison of prefrontal cortex, striatum, and hippocampus among matched tetrads of controls with subjects diagnosed with schizophrenia, bipolar or major depressive disorder. Transl Psychiatry. 2019;9:151. 31123247 10.1038/s41398-019-0492-8 PMC6533277

[53] Groos D, Zheng F, Rauh M, Quinger B, Kornhuber J, Müller CP, et al. Chronic antipsychotic treatment reverses GIRK current suppression in dopaminergic neurons, loss of accumbal LTD, and behavioral sensitization in a mouse model of amphetamine psychosis. J Psychopharmacol. 2019;33:74–85. 10.1177/0269881118812235 30488738

[54] Amato D, Natesan S, Yavich L, Kapur S, Müller CP. Dynamic regulation of dopamine and serotonin responses to salient stimuli during chronic haloperidol treatment. Int J Neuropsychopharm. 2011;14:1327–39. 10.1017/S1461145711000010 21281560

[55] Lustig C, Meck WH. Chronic treatment with haloperidol induces deficits in working memory and feedback effects of interval timing. Brain Cogn. 2005;58:9–16. 15878723 10.1016/j.bandc.2004.09.005

[56] Müller CP, Kalinichenko LS, Tiesel J, Witt M, Stöckl T, Sprenger E, et al. Paradoxical antidepressant effects of alcohol are related to acid sphingomyelinase and its control of sphingolipid homeostasis. Acta Neuropathologica. 2017;133:463–83. 28000031 10.1007/s00401-016-1658-6 PMC5325869

[57] Kalinichenko LS, Mühle C, Jia T, Anderheiden F, Datz M, Eberle AL, et al. Neutral sphingomyelinase mediates the comorbidity trias of alcohol abuse, major depression and bone defects. Mol Psychiatry. 2021;26:7403–16. 34584229 10.1038/s41380-021-01304-w PMC8872992

[58] Quednow BB, Kühn KU, Mössner R, Schwab SG, Schuhmacher A, Maier W, et al. Sensorimotor gating of schizophrenia patients is influenced by 5-HT2A receptor polymorphisms. Biol Psychiatry. 2008;64:434–7. 18420180 10.1016/j.biopsych.2008.02.019

[59] Schizophrenia Working Group (SWG) of the Psychiatric Genomics Consortium. Biological insights from 108 schizophrenia-associated genetic loci. Nature. 2014;511:421–7. 25056061 10.1038/nature13595 PMC4112379

[60] Rogdaki M, Devroye C, Ciampoli M, Veronese M, Ashok AH, McCutcheon RA, et al. Striatal dopaminergic alterations in individuals with copy number variants at the 22q11.2 genetic locus and their implications for psychosis risk: a [18F]-DOPA PET study. Mol Psychiatry. 2023;28:1995–2006. 33981004 10.1038/s41380-021-01108-y PMC10575769

[61] Schneider M, Levant B, Reichel M, Gulbins E, Kornhuber J, Müller CP. Lipids in psychiatric disorders and preventive medicine. Neurosci Biobehav Rev. 2017;76:336–62. 27317860 10.1016/j.neubiorev.2016.06.002

[62] Choi BJ, Park MH, Park KH, Han WH, Yoon HJ, Jung HY, et al. Immunotherapy targeting plasma ASM is protective in a mouse model of Alzheimer’s disease. Nat Commun. 2023;14:1631. 36959217 10.1038/s41467-023-37316-z PMC10036484

[63] Kornhuber J, Müller CP, Becker AK, Reichel M, Gulbins E. The ceramide system as a novel antidepressant target. Trends Pharmacol Sci. 2014;35:293–304. 24793541 10.1016/j.tips.2014.04.003

[64] Zoicas I, Huber SE, Kalinichenko LS, Gulbins E, Müller CP, Kornhuber J. Ceramides affect alcohol consumption and depressive-like and anxiety-like behavior in a brain region- and ceramide species-specific way in male mice. Addiction Biol. 2020;25:e12847. 10.1111/adb.12847 31828921

[65] Knight S, McCutcheon R, Dwir D, Grace AA, O’Daly O, McGuire P, et al. Hippocampal circuit dysfunction in psychosis. Transl Psychiatry. 2022;12:344. 36008395 10.1038/s41398-022-02115-5 PMC9411597

[66] Zoicas I, Reichel M, Gulbins E, Kornhuber J. Role of acid sphingomyelinase in the regulation of social behavior and memory. PLoS ONE. 2016;11:e0162498. 27598773 10.1371/journal.pone.0162498 PMC5012580

[67] Sigurdsson T, Stark KL, Karayiorgou M, Gogos JA, Gordon JA. Impaired hippocampal-prefrontal synchrony in a genetic mouse model of schizophrenia. Nature. 2010;464:763–7. 20360742 10.1038/nature08855 PMC2864584

[68] Jaffe AE, Gao Y, Deep-Soboslay A, Tao R, Hyde TM, Weinberger DR, et al. Mapping DNA methylation across development, genotype and schizophrenia in the human frontal cortex. Nat Neurosci. 2016;19:40–7. 26619358 10.1038/nn.4181 PMC4783176

[69] Horrobin DF, Glen AIM, Vaddadi K. The membrane hypothesis of schizophrenia. Schizophrenia Res. 1994;13:195–207. 10.1016/0920-9964(94)90043-4 7841132

[70] Wood PL. Targeted lipidomics and metabolomics evaluations of cortical neuronal stress in schizophrenia. Schizophr Res. 2019;212:107–12. 31434624 10.1016/j.schres.2019.08.003

[71] Zoicas I, Schumacher F, Kleuser B, Reichel M, Gulbins E, Fejtova A, et al. Forebrain-specific overexpression of acid sphingomyelinase induces depressive-like symptoms in mice. Cells. 2020;9:1244. 32443534 10.3390/cells9051244 PMC7290754

[72] Kornhuber J, Tripal P, Gulbins E, Muehlbacher M. Functional inhibitors of acid sphingomyelinase (FIASMAs). Handbook Exp Pharmacol. 2013;215:169–86. 10.1007/978-3-7091-1368-4_9 23579455

[73] Tischbirek CH, Wenzel EM, Zheng F, Huth T, Amato D, Trapp S, et al. Use-dependent inhibition of synaptic transmission by the secretion of intravesicularly accumulated antipsychotic drugs. Neuron. 2012;74:830–44. 22681688 10.1016/j.neuron.2012.04.019

[74] Kornhuber J, Muehlbacher M, Trapp S, Pechmann S, Friedl A, Reichel M, et al. Identification of novel functional inhibitors of acid sphingomyelinase. PLoS ONE. 2011;6:e23852. 21909365 10.1371/journal.pone.0023852 PMC3166082

[75] Kapur S, Vanderspek SC, Brownlee BA, Nobrega JN. Antipsychotic dosing in preclinical models is often unrepresentative of the clinical condition: a suggested solution based on in vivo occupancy. J Pharmacol Exp Therap. 2003;305:625–31. 12606608 10.1124/jpet.102.046987

[76] Lieberman JA, Stroup TS, McEvoy JP, Swartz MS, Rosenheck RA, Perkins DO, et al. Effectiveness of antipsychotic drugs in patients with chronic schizophrenia. NJEM. 2005;353:1209–23. 10.1056/NEJMoa051688 16172203

[77] Stroup TS, Lieberman JA, McEvoy JP, Swartz MS, Davis SM, Rosenheck RA, et al. Effectiveness of olanzapine, quetiapine, risperidone, and ziprasidone in patients with chronic schizophrenia following discontinuation of a previous atypical antipsychotic. Am J Psychiatry. 2006;163:611–22. 16585435 10.1176/ajp.2006.163.4.611

[78] Amato D. Serotonin in antipsychotic drugs action. Behav Brain Res. 2015;277:125–35. 25078293 10.1016/j.bbr.2014.07.025

[79] Quednow BB, Geyer MA, Halberstadt AL. Serotonin and schizophrenia. In: Müller CP, Cunningham KA, editors. Handbook of the behavioral neurobiology of serotonin. London: Academic Press; 2020. pp. 711–43.

[80] Kalinichenko LS, Hammad L, Reichel M, Kohl Z, Gulbins E, Kornhuber J, et al. Acid sphingomyelinase controls dopamine activity and responses to appetitive stimuli in mice. Brain Res Bull. 2019;146:310–9. 30716394 10.1016/j.brainresbull.2019.01.026

[81] Karl T, Duffy L, O’Brien E, Matsumoto I, Dedova I. Behavioural effects of chronic haloperidol and risperidone treatment in rats. Behav Brain Res. 2006;171:286–94. 16697060 10.1016/j.bbr.2006.04.004

[82] Falkai P, Steiner J, Malchow B, Shariati J, Knaus A, Bernstein HG, et al. Oligodendrocyte and interneuron density in hippocampal subfields in schizophrenia and association of oligodendrocyte number with cognitive deficits. Front Cell Neurosci. 2016;10:78. 27065804 10.3389/fncel.2016.00078 PMC4811909

[83] Rogério Ferreira F, Barros de Moura NS, Hassib L, Pombo TR. Resveratrol ameliorates the effect of maternal immune activation associated with schizophrenia in adulthood offspring. Neurosci Lett. 2020;734:135100. 32473196 10.1016/j.neulet.2020.135100

[84] Narla ST, Lee YW, Benson CA, Sarder P, Brennand KJ, Stachowiak EK, et al. Common developmental genome deprogramming in schizophrenia—role of Integrative Nuclear FGFR1 Signaling (INFS). Schizophr Res. 2017;185:17–32. 28094170 10.1016/j.schres.2016.12.012 PMC5507209

[85] Stachowiak EK, Benson CA, Narla ST, Dimitri A, Bayona Chuye LE, Dhiman S, et al. Cerebral organoids reveal early cortical maldevelopment in schizophrenia-computational anatomy and genomics, role of FGFR1. Transl Psychiatry. 2017;7:6. 30446636 10.1038/s41398-017-0054-x PMC5802550

[86] Prata DP, Costa-Neves B, Cosme G, Vassos E. Unravelling the genetic basis of schizophrenia and bipolar disorder with GWAS: a systematic review. J Psychiatr Res. 2019;114:178–207. 31096178 10.1016/j.jpsychires.2019.04.007

[87] Jia P, Wang L, Fanous AH, Pato CN, Edwards TL, International Schizophrenia Consortium, et al. Network-assisted investigation of combined causal signals from genome-wide association studies in schizophrenia. PLoS Comput Biol. 2012;8:e1002587. 22792057 10.1371/journal.pcbi.1002587 PMC3390381

[88] Lu C, Dong L, Zhou H, Li Q, Huang G, Bai SJ, et al. G-protein-coupled receptor Gpr17 regulates oligodendrocyte differentiation in response to lysolecithin-induced demyelination. Sci Rep. 2018;8:4502. 29540737 10.1038/s41598-018-22452-0 PMC5852120

[89] Wang Q, et al. Increased co-expression of genes harboring the damaging de novo mutations in Chinese schizophrenic patients during prenatal development. Sci Rep. 2015;5:18209. 26666178 10.1038/srep18209 PMC4678883

[90] Xia M, Broek JAC, Jouroukhin Y, Schoenfelder J, Abazyan S, Jaaro-Peled H, et al. Cell type-specific effects of mutant DISC1: a proteomics study. Mol Neuropsychiatry. 2016;2:28–36. 27606318 10.1159/000444587 PMC4996005

[91] Peter-Ross EM. Molecular hypotheses to explain the shared pathways and underlying pathobiological causes in catatonia and in catatonic presentations in neuropsychiatric disorders. Med Hypotheses. 2018;113:54–64. 29523295 10.1016/j.mehy.2018.02.009

[92] Takata A, Ionita-Laza I, Gogos JA, Xu B, Karayiorgou M. De novo synonymous mutations in regulatory elements contribute to the genetic etiology of Autism and Schizophrenia. Neuron. 2016;89:940–7. 26938441 10.1016/j.neuron.2016.02.024 PMC4793939

[93] Bhuiyan P, Sun Z, Khan MA, Hossain MA, Rahman MH, Qian Y. System biology approaches to identify hub genes linked with ECM organization and inflammatory signaling pathways in schizophrenia pathogenesis. Heliyon. 2024;10:e25191. 38322840 10.1016/j.heliyon.2024.e25191 PMC10844262

[94] Uwai Y, Honjo H, Iwamoto K. Interaction and transport of kynurenic acid via human organic anion transporters hOAT1 and hOAT3. Pharmacol Res. 2012;65:254–60. 22108572 10.1016/j.phrs.2011.11.003

[95] Wang Q, Li M, Yang Z, Hu X, Wu HM, Ni P, et al. Genome-wide association study identified six loci associated with adverse drug reactions to aripiprazole in schizophrenia patients. Schizophrenia. 2023;9:44. 37491364 10.1038/s41537-023-00369-6 PMC10368716

[96] Johansson AS, Owe-Larsson B, Hetta J, Lundkvist GB. Altered circadian clock gene expression in patients with schizophrenia. Schizophr Res. 2016;174:17–23. 27132483 10.1016/j.schres.2016.04.029

[97] Lee SB, Park J, Kwak Y, Park YU, Nhung TTM, Suh BK, et al. Disrupted-in-schizophrenia 1 enhances the quality of circadian rhythm by stabilizing BMAL1. Transl Psychiatry. 2021;11:110. 33542182 10.1038/s41398-021-01212-1 PMC7862247

[98] Hass J, Walton E, Kirsten H, Liu J, Priebe L, Wolf C, et al. A genome-wide association study suggests novel loci associated with a schizophrenia-related brain-based phenotype. PLoS ONE. 2013;8:e64872. 23805179 10.1371/journal.pone.0064872 PMC3689744

[99] van Kruining D, Luo Q, van Echten-Deckert G, Mielke MM, Bowman A, Ellis S, et al. Sphingolipids as prognostic biomarkers of neurodegeneration, neuroinflammation, and psychiatric diseases and their emerging role in lipidomic investigation methods. Adv Drug Delivery Rev. 2020;159:232–44. 10.1016/j.addr.2020.04.009 PMC7665829 32360155

