# Supplementary material for: Acid sphingomyelinase activity suggests a new antipsychotic pharmaco-treatment strategy for schizophrenia
Source: Mol Psychiatry. 2025 Jan 17;30(7):2891–906. doi: 10.1038/s41380-025-02893-6 (PMC12185314; doi:10.1038/s41380-025-02893-6)
Supplement: Supplementary file 1 — Supplemental material [file 41380_2025_2893_MOESM1_ESM.pptx]

## Slide 1
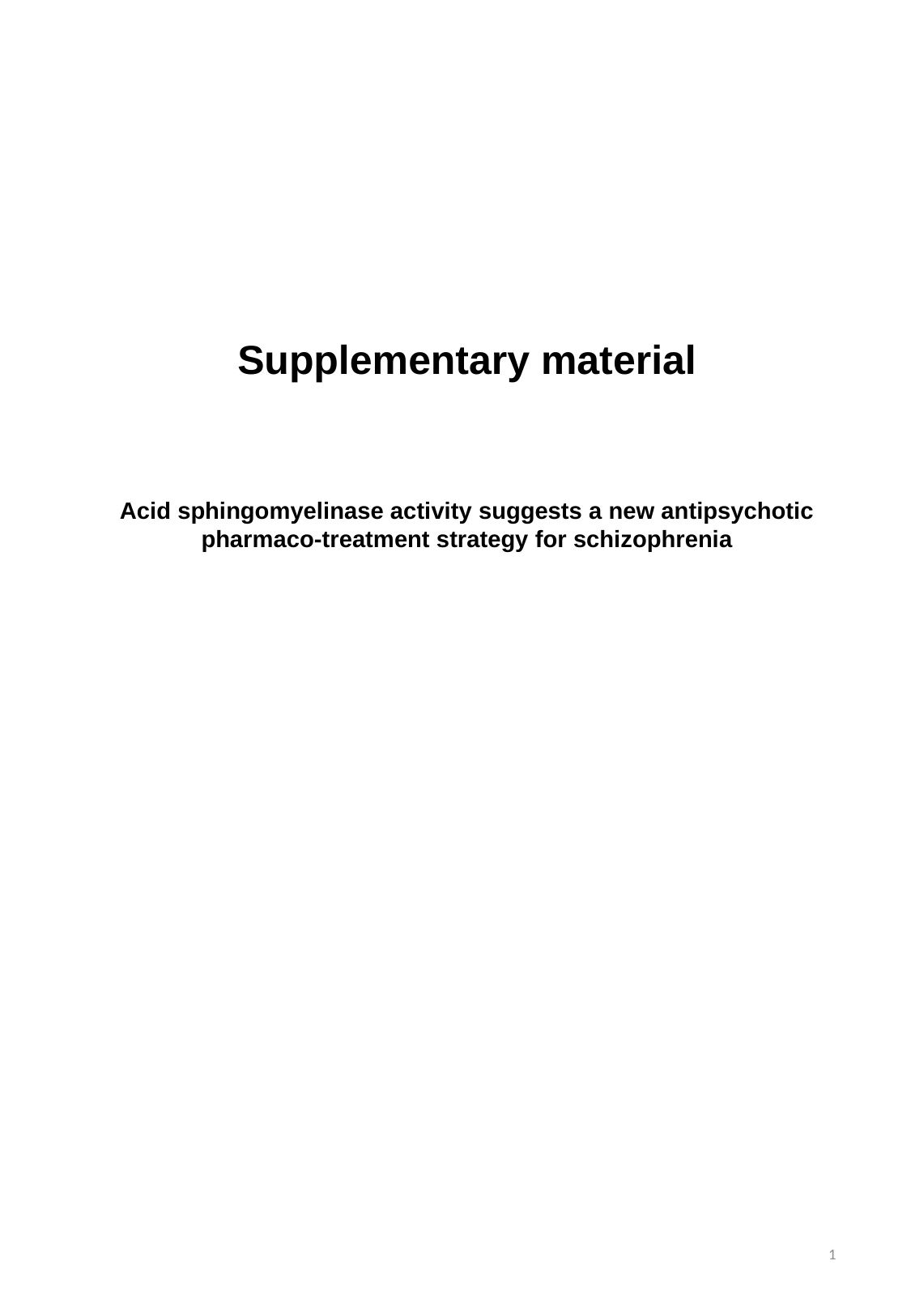

Supplementary material
Acid sphingomyelinase activity suggests a new antipsychotic pharmaco-treatment strategy for schizophrenia
1

## Slide 2
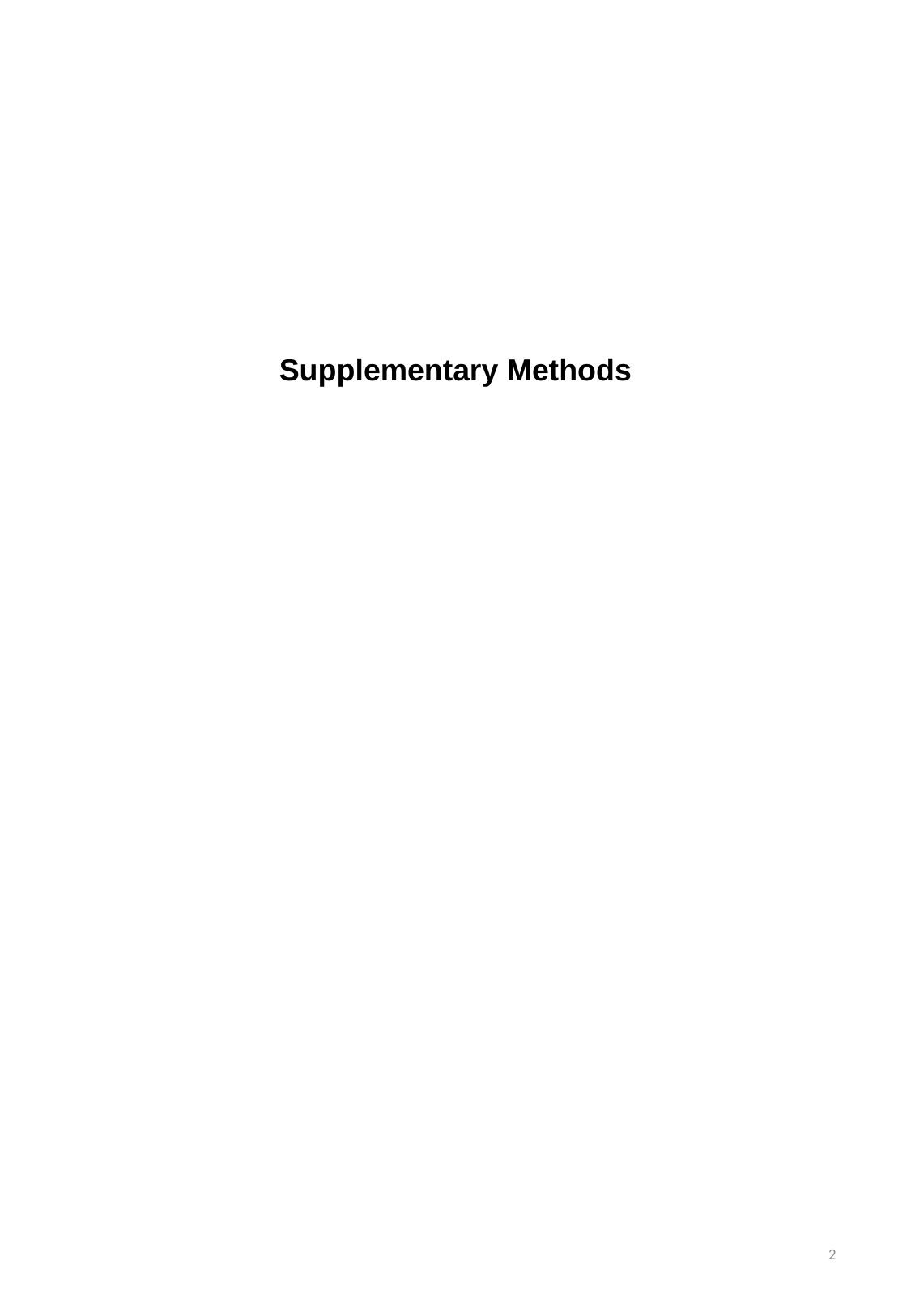

Supplementary Methods
2

## Slide 3
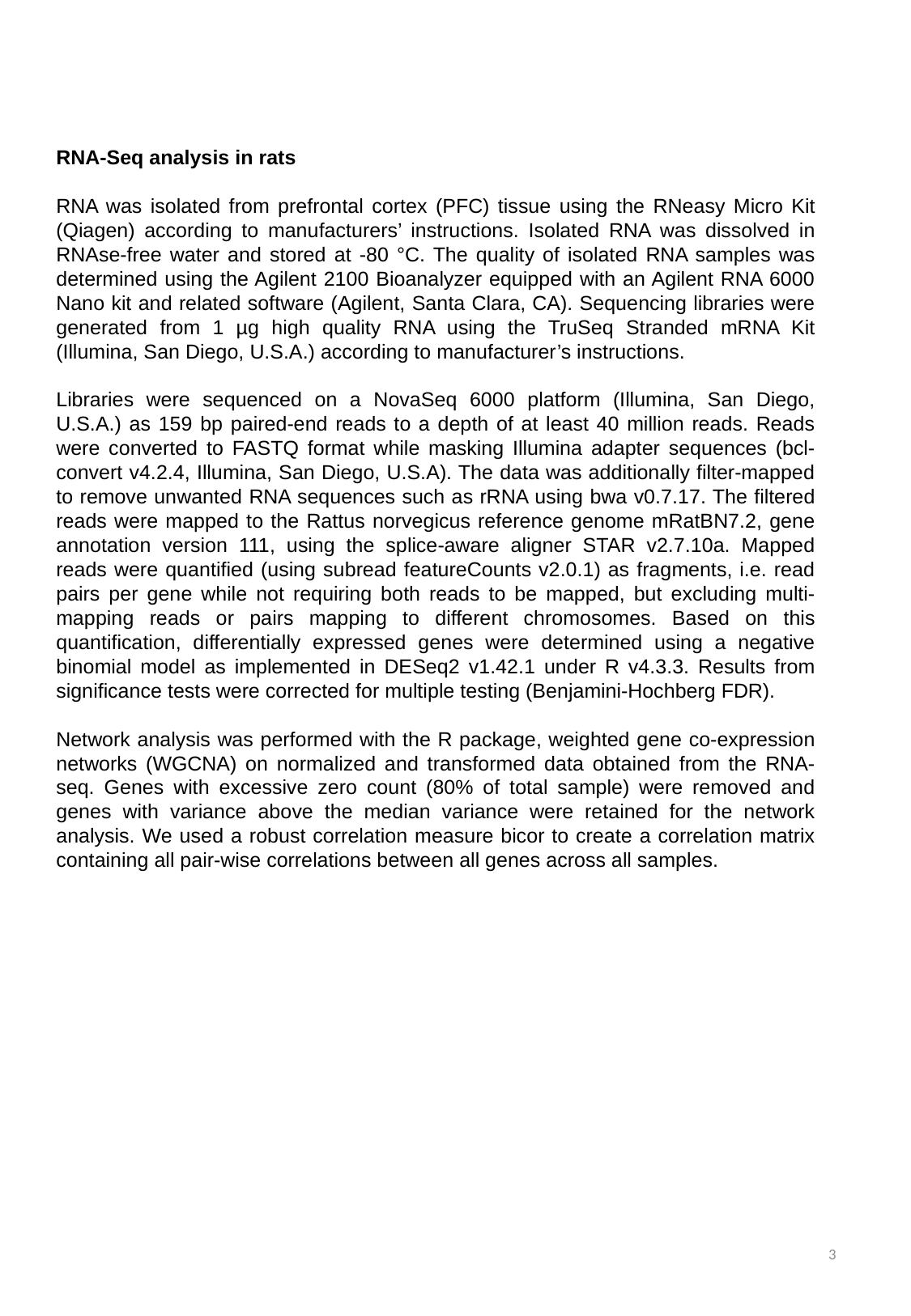

RNA-Seq analysis in rats
RNA was isolated from prefrontal cortex (PFC) tissue using the RNeasy Micro Kit (Qiagen) according to manufacturers’ instructions. Isolated RNA was dissolved in RNAse-free water and stored at -80 °C. The quality of isolated RNA samples was determined using the Agilent 2100 Bioanalyzer equipped with an Agilent RNA 6000 Nano kit and related software (Agilent, Santa Clara, CA). Sequencing libraries were generated from 1 µg high quality RNA using the TruSeq Stranded mRNA Kit (Illumina, San Diego, U.S.A.) according to manufacturer’s instructions.
Libraries were sequenced on a NovaSeq 6000 platform (Illumina, San Diego, U.S.A.) as 159 bp paired-end reads to a depth of at least 40 million reads. Reads were converted to FASTQ format while masking Illumina adapter sequences (bcl-convert v4.2.4, Illumina, San Diego, U.S.A). The data was additionally filter-mapped to remove unwanted RNA sequences such as rRNA using bwa v0.7.17. The filtered reads were mapped to the Rattus norvegicus reference genome mRatBN7.2, gene annotation version 111, using the splice-aware aligner STAR v2.7.10a. Mapped reads were quantified (using subread featureCounts v2.0.1) as fragments, i.e. read pairs per gene while not requiring both reads to be mapped, but excluding multi-mapping reads or pairs mapping to different chromosomes. Based on this quantification, differentially expressed genes were determined using a negative binomial model as implemented in DESeq2 v1.42.1 under R v4.3.3. Results from significance tests were corrected for multiple testing (Benjamini-Hochberg FDR).
Network analysis was performed with the R package, weighted gene co-expression networks (WGCNA) on normalized and transformed data obtained from the RNA-seq. Genes with excessive zero count (80% of total sample) were removed and genes with variance above the median variance were retained for the network analysis. We used a robust correlation measure bicor to create a correlation matrix containing all pair-wise correlations between all genes across all samples.
3

## Slide 4
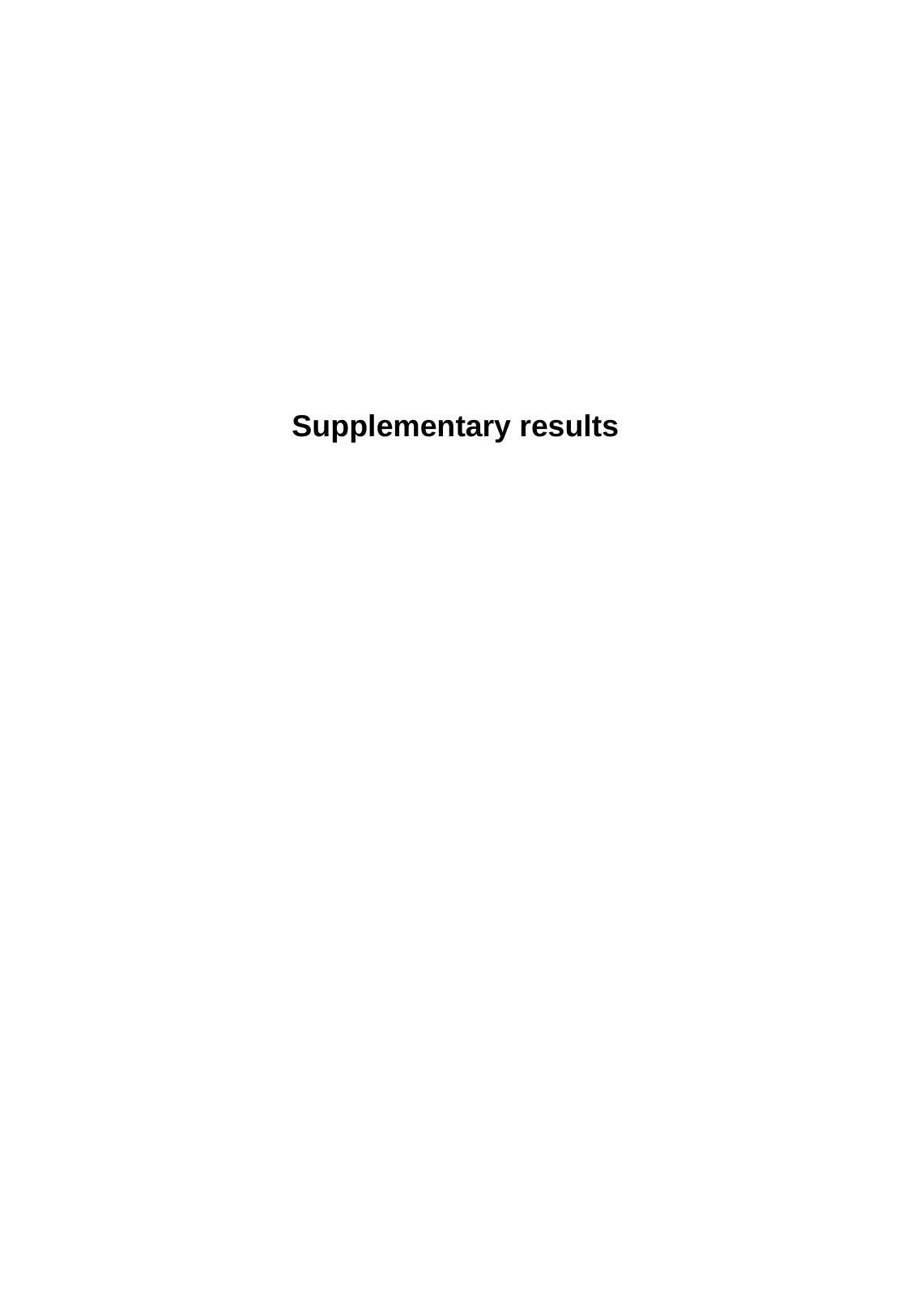

Supplementary results

## Slide 5
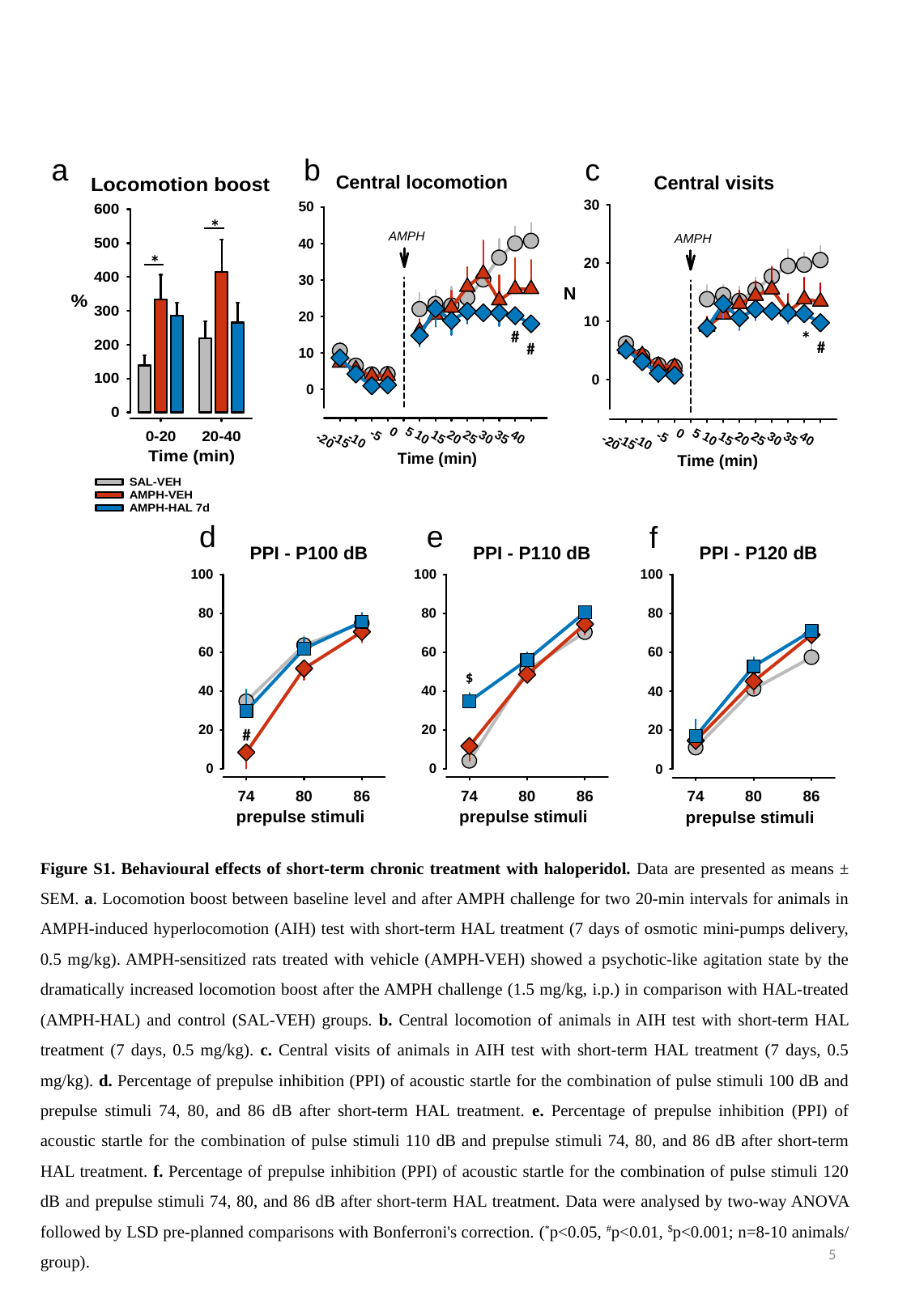

a
b
c
#
#
*
*
*
#
#
$
d
e
f
Figure S1. Behavioural effects of short-term chronic treatment with haloperidol. Data are presented as means ± SEM. a. Locomotion boost between baseline level and after AMPH challenge for two 20-min intervals for animals in AMPH-induced hyperlocomotion (AIH) test with short-term HAL treatment (7 days of osmotic mini-pumps delivery, 0.5 mg/kg). AMPH-sensitized rats treated with vehicle (AMPH-VEH) showed a psychotic-like agitation state by the dramatically increased locomotion boost after the AMPH challenge (1.5 mg/kg, i.p.) in comparison with HAL-treated (AMPH-HAL) and control (SAL-VEH) groups. b. Central locomotion of animals in AIH test with short-term HAL treatment (7 days, 0.5 mg/kg). c. Central visits of animals in AIH test with short-term HAL treatment (7 days, 0.5 mg/kg). d. Percentage of prepulse inhibition (PPI) of acoustic startle for the combination of pulse stimuli 100 dB and prepulse stimuli 74, 80, and 86 dB after short-term HAL treatment. e. Percentage of prepulse inhibition (PPI) of acoustic startle for the combination of pulse stimuli 110 dB and prepulse stimuli 74, 80, and 86 dB after short-term HAL treatment. f. Percentage of prepulse inhibition (PPI) of acoustic startle for the combination of pulse stimuli 120 dB and prepulse stimuli 74, 80, and 86 dB after short-term HAL treatment. Data were analysed by two-way ANOVA followed by LSD pre-planned comparisons with Bonferroni's correction. (*p<0.05, #p<0.01, $p<0.001; n=8-10 animals/ group).
5

## Slide 6
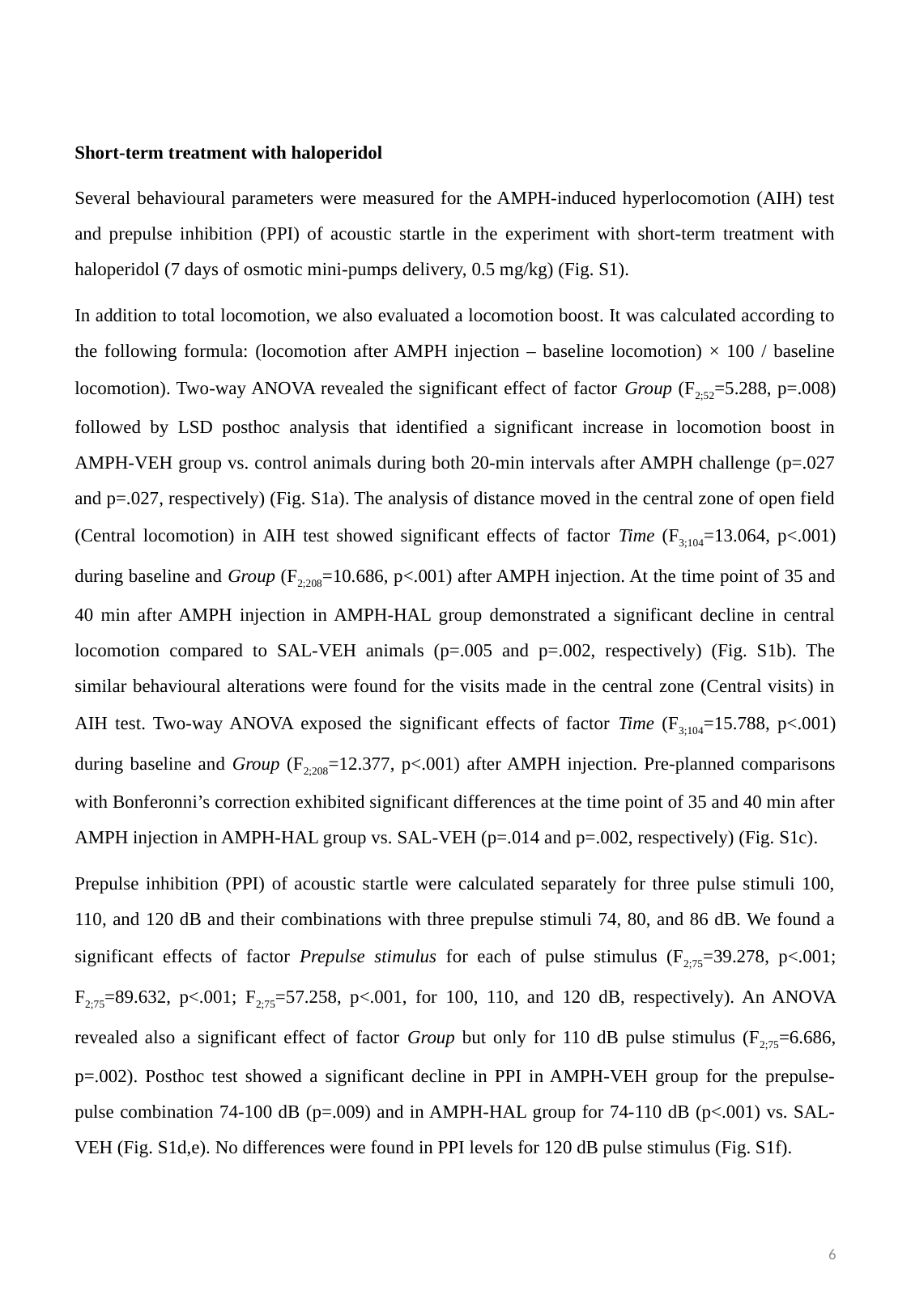

Short-term treatment with haloperidol
Several behavioural parameters were measured for the AMPH-induced hyperlocomotion (AIH) test and prepulse inhibition (PPI) of acoustic startle in the experiment with short-term treatment with haloperidol (7 days of osmotic mini-pumps delivery, 0.5 mg/kg) (Fig. S1).
In addition to total locomotion, we also evaluated a locomotion boost. It was calculated according to the following formula: (locomotion after AMPH injection – baseline locomotion) × 100 / baseline locomotion). Two-way ANOVA revealed the significant effect of factor Group (F2;52=5.288, p=.008) followed by LSD posthoc analysis that identified a significant increase in locomotion boost in AMPH-VEH group vs. control animals during both 20-min intervals after AMPH challenge (p=.027 and p=.027, respectively) (Fig. S1a). The analysis of distance moved in the central zone of open field (Central locomotion) in AIH test showed significant effects of factor Time (F3;104=13.064, p<.001) during baseline and Group (F2;208=10.686, p<.001) after AMPH injection. At the time point of 35 and 40 min after AMPH injection in AMPH-HAL group demonstrated a significant decline in central locomotion compared to SAL-VEH animals (p=.005 and p=.002, respectively) (Fig. S1b). The similar behavioural alterations were found for the visits made in the central zone (Central visits) in AIH test. Two-way ANOVA exposed the significant effects of factor Time (F3;104=15.788, p<.001) during baseline and Group (F2;208=12.377, p<.001) after AMPH injection. Pre-planned comparisons with Bonferonni’s correction exhibited significant differences at the time point of 35 and 40 min after AMPH injection in AMPH-HAL group vs. SAL-VEH (p=.014 and p=.002, respectively) (Fig. S1c).
Prepulse inhibition (PPI) of acoustic startle were calculated separately for three pulse stimuli 100, 110, and 120 dB and their combinations with three prepulse stimuli 74, 80, and 86 dB. We found a significant effects of factor Prepulse stimulus for each of pulse stimulus (F2;75=39.278, p<.001; F2;75=89.632, p<.001; F2;75=57.258, p<.001, for 100, 110, and 120 dB, respectively). An ANOVA revealed also a significant effect of factor Group but only for 110 dB pulse stimulus (F2;75=6.686, p=.002). Posthoc test showed a significant decline in PPI in AMPH-VEH group for the prepulse-pulse combination 74-100 dB (p=.009) and in AMPH-HAL group for 74-110 dB (p<.001) vs. SAL-VEH (Fig. S1d,e). No differences were found in PPI levels for 120 dB pulse stimulus (Fig. S1f).
6

## Slide 7
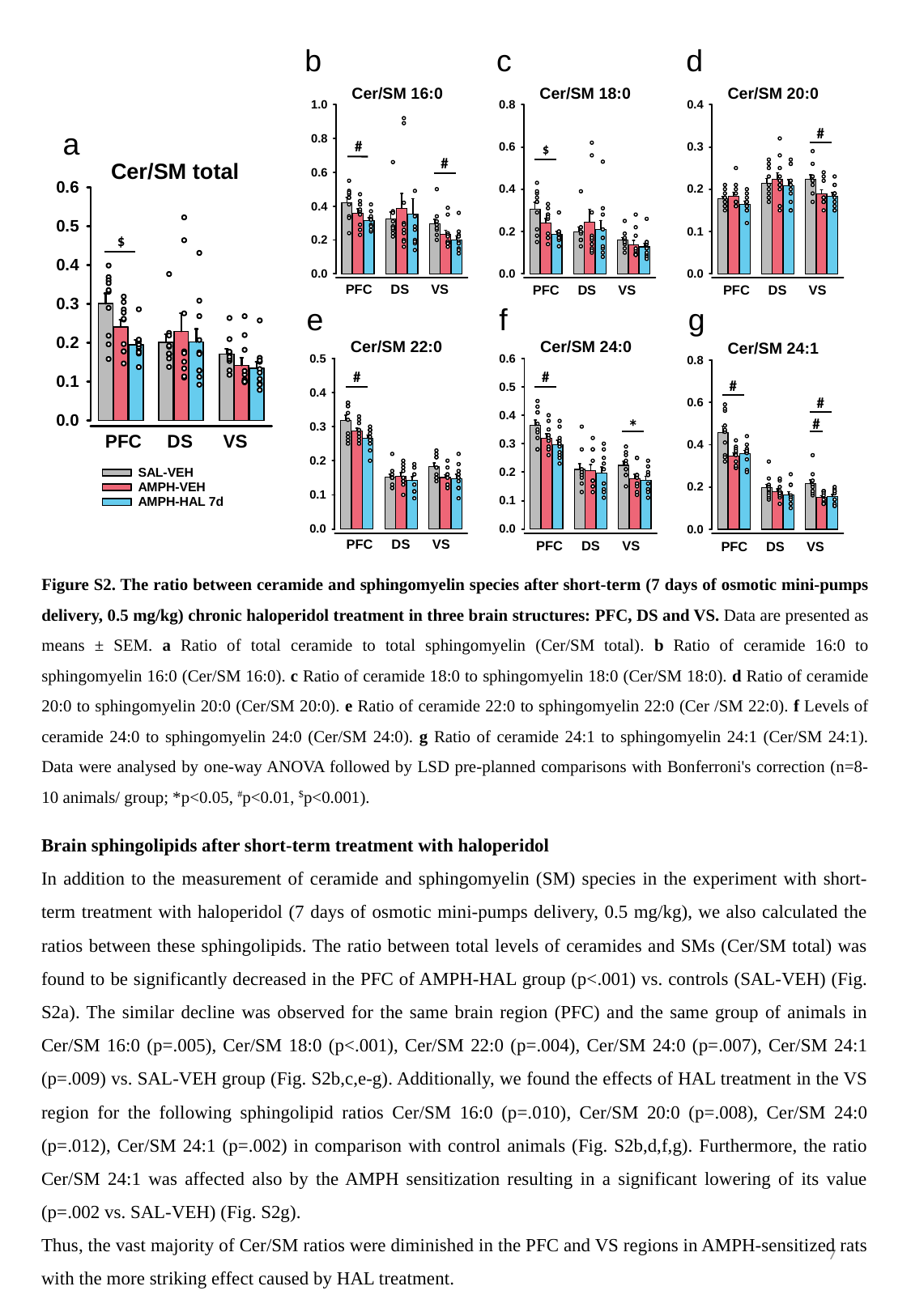

b
c
a
d
e
f
g
#
#
$
#
$
#
#
*
#
#
#
Figure S2. The ratio between ceramide and sphingomyelin species after short-term (7 days of osmotic mini-pumps delivery, 0.5 mg/kg) chronic haloperidol treatment in three brain structures: PFC, DS and VS. Data are presented as means ± SEM. a Ratio of total ceramide to total sphingomyelin (Cer/SM total). b Ratio of ceramide 16:0 to sphingomyelin 16:0 (Cer/SM 16:0). c Ratio of ceramide 18:0 to sphingomyelin 18:0 (Cer/SM 18:0). d Ratio of ceramide 20:0 to sphingomyelin 20:0 (Cer/SM 20:0). e Ratio of ceramide 22:0 to sphingomyelin 22:0 (Cer /SM 22:0). f Levels of ceramide 24:0 to sphingomyelin 24:0 (Cer/SM 24:0). g Ratio of ceramide 24:1 to sphingomyelin 24:1 (Cer/SM 24:1). Data were analysed by one-way ANOVA followed by LSD pre-planned comparisons with Bonferroni's correction (n=8-10 animals/ group; *p<0.05, #p<0.01, $p<0.001).
Brain sphingolipids after short-term treatment with haloperidol
In addition to the measurement of ceramide and sphingomyelin (SM) species in the experiment with short-term treatment with haloperidol (7 days of osmotic mini-pumps delivery, 0.5 mg/kg), we also calculated the ratios between these sphingolipids. The ratio between total levels of ceramides and SMs (Cer/SM total) was found to be significantly decreased in the PFC of AMPH-HAL group (p<.001) vs. controls (SAL-VEH) (Fig. S2a). The similar decline was observed for the same brain region (PFC) and the same group of animals in Cer/SM 16:0 (p=.005), Cer/SM 18:0 (p<.001), Cer/SM 22:0 (p=.004), Cer/SM 24:0 (p=.007), Cer/SM 24:1 (p=.009) vs. SAL-VEH group (Fig. S2b,c,e-g). Additionally, we found the effects of HAL treatment in the VS region for the following sphingolipid ratios Cer/SM 16:0 (p=.010), Cer/SM 20:0 (p=.008), Cer/SM 24:0 (p=.012), Cer/SM 24:1 (p=.002) in comparison with control animals (Fig. S2b,d,f,g). Furthermore, the ratio Cer/SM 24:1 was affected also by the AMPH sensitization resulting in a significant lowering of its value (p=.002 vs. SAL-VEH) (Fig. S2g).
Thus, the vast majority of Cer/SM ratios were diminished in the PFC and VS regions in AMPH-sensitized rats with the more striking effect caused by HAL treatment.
7

## Slide 8
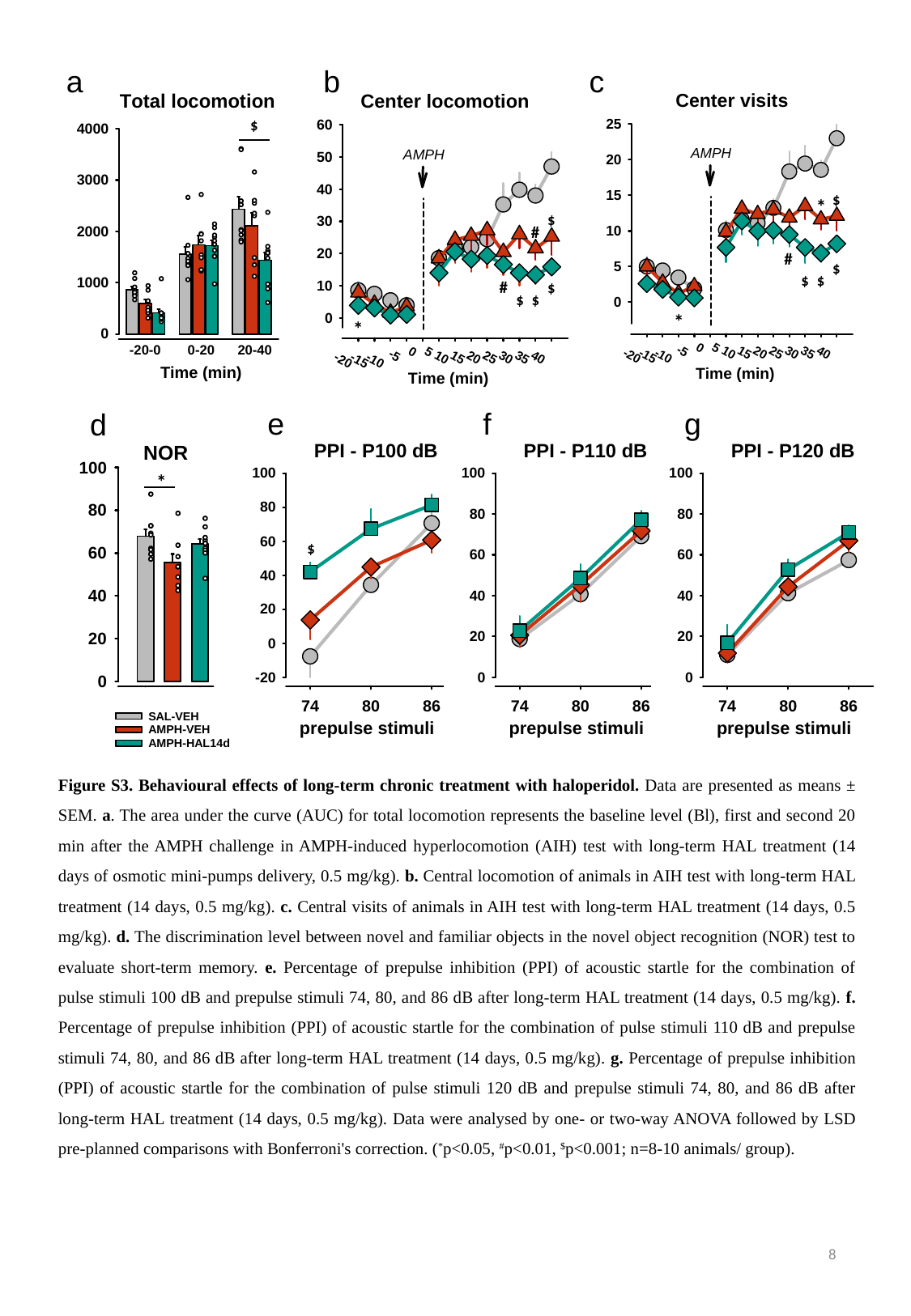

a
c
b
$
#
#
$
$
$
*
$
*
#
$
$
$
*
$
-20-0 0-20 20-40
e
f
d
g
*
$
Figure S3. Behavioural effects of long-term chronic treatment with haloperidol. Data are presented as means ± SEM. a. The area under the curve (AUC) for total locomotion represents the baseline level (Bl), first and second 20 min after the AMPH challenge in AMPH-induced hyperlocomotion (AIH) test with long-term HAL treatment (14 days of osmotic mini-pumps delivery, 0.5 mg/kg). b. Central locomotion of animals in AIH test with long-term HAL treatment (14 days, 0.5 mg/kg). c. Central visits of animals in AIH test with long-term HAL treatment (14 days, 0.5 mg/kg). d. The discrimination level between novel and familiar objects in the novel object recognition (NOR) test to evaluate short-term memory. e. Percentage of prepulse inhibition (PPI) of acoustic startle for the combination of pulse stimuli 100 dB and prepulse stimuli 74, 80, and 86 dB after long-term HAL treatment (14 days, 0.5 mg/kg). f. Percentage of prepulse inhibition (PPI) of acoustic startle for the combination of pulse stimuli 110 dB and prepulse stimuli 74, 80, and 86 dB after long-term HAL treatment (14 days, 0.5 mg/kg). g. Percentage of prepulse inhibition (PPI) of acoustic startle for the combination of pulse stimuli 120 dB and prepulse stimuli 74, 80, and 86 dB after long-term HAL treatment (14 days, 0.5 mg/kg). Data were analysed by one- or two-way ANOVA followed by LSD pre-planned comparisons with Bonferroni's correction. (*p<0.05, #p<0.01, $p<0.001; n=8-10 animals/ group).
8

## Slide 9
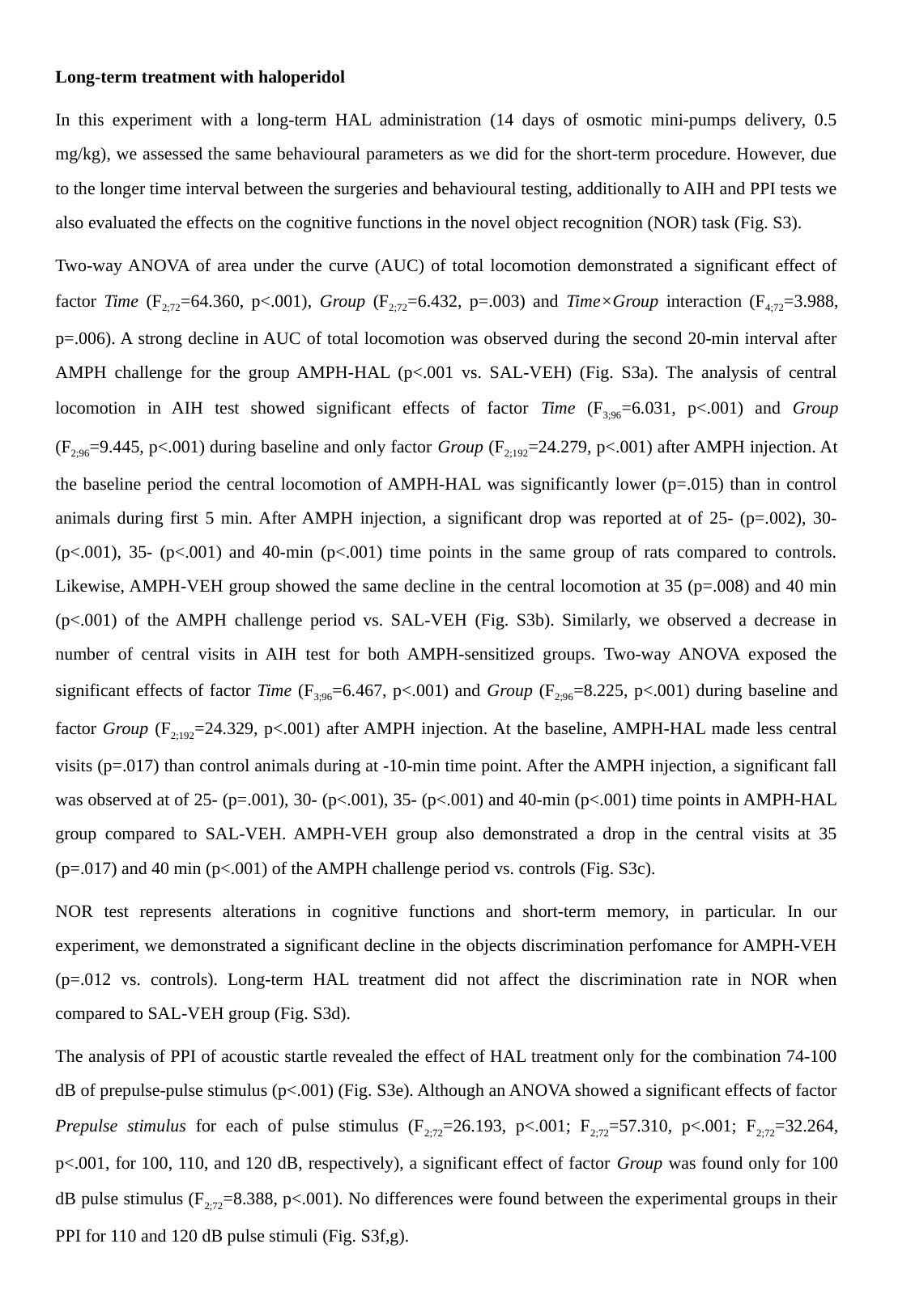

Long-term treatment with haloperidol
In this experiment with a long-term HAL administration (14 days of osmotic mini-pumps delivery, 0.5 mg/kg), we assessed the same behavioural parameters as we did for the short-term procedure. However, due to the longer time interval between the surgeries and behavioural testing, additionally to AIH and PPI tests we also evaluated the effects on the cognitive functions in the novel object recognition (NOR) task (Fig. S3).
Two-way ANOVA of area under the curve (AUC) of total locomotion demonstrated a significant effect of factor Time (F2;72=64.360, p<.001), Group (F2;72=6.432, p=.003) and Time×Group interaction (F4;72=3.988, p=.006). A strong decline in AUC of total locomotion was observed during the second 20-min interval after AMPH challenge for the group AMPH-HAL (p<.001 vs. SAL-VEH) (Fig. S3a). The analysis of central locomotion in AIH test showed significant effects of factor Time (F3;96=6.031, p<.001) and Group (F2;96=9.445, p<.001) during baseline and only factor Group (F2;192=24.279, p<.001) after AMPH injection. At the baseline period the central locomotion of AMPH-HAL was significantly lower (p=.015) than in control animals during first 5 min. After AMPH injection, a significant drop was reported at of 25- (p=.002), 30- (p<.001), 35- (p<.001) and 40-min (p<.001) time points in the same group of rats compared to controls. Likewise, AMPH-VEH group showed the same decline in the central locomotion at 35 (p=.008) and 40 min (p<.001) of the AMPH challenge period vs. SAL-VEH (Fig. S3b). Similarly, we observed a decrease in number of central visits in AIH test for both AMPH-sensitized groups. Two-way ANOVA exposed the significant effects of factor Time (F3;96=6.467, p<.001) and Group (F2;96=8.225, p<.001) during baseline and factor Group (F2;192=24.329, p<.001) after AMPH injection. At the baseline, AMPH-HAL made less central visits (p=.017) than control animals during at -10-min time point. After the AMPH injection, a significant fall was observed at of 25- (p=.001), 30- (p<.001), 35- (p<.001) and 40-min (p<.001) time points in AMPH-HAL group compared to SAL-VEH. AMPH-VEH group also demonstrated a drop in the central visits at 35 (p=.017) and 40 min (p<.001) of the AMPH challenge period vs. controls (Fig. S3c).
NOR test represents alterations in cognitive functions and short-term memory, in particular. In our experiment, we demonstrated a significant decline in the objects discrimination perfomance for AMPH-VEH (p=.012 vs. controls). Long-term HAL treatment did not affect the discrimination rate in NOR when compared to SAL-VEH group (Fig. S3d).
The analysis of PPI of acoustic startle revealed the effect of HAL treatment only for the combination 74-100 dB of prepulse-pulse stimulus (p<.001) (Fig. S3e). Although an ANOVA showed a significant effects of factor Prepulse stimulus for each of pulse stimulus (F2;72=26.193, p<.001; F2;72=57.310, p<.001; F2;72=32.264, p<.001, for 100, 110, and 120 dB, respectively), a significant effect of factor Group was found only for 100 dB pulse stimulus (F2;72=8.388, p<.001). No differences were found between the experimental groups in their PPI for 110 and 120 dB pulse stimuli (Fig. S3f,g).

## Slide 10
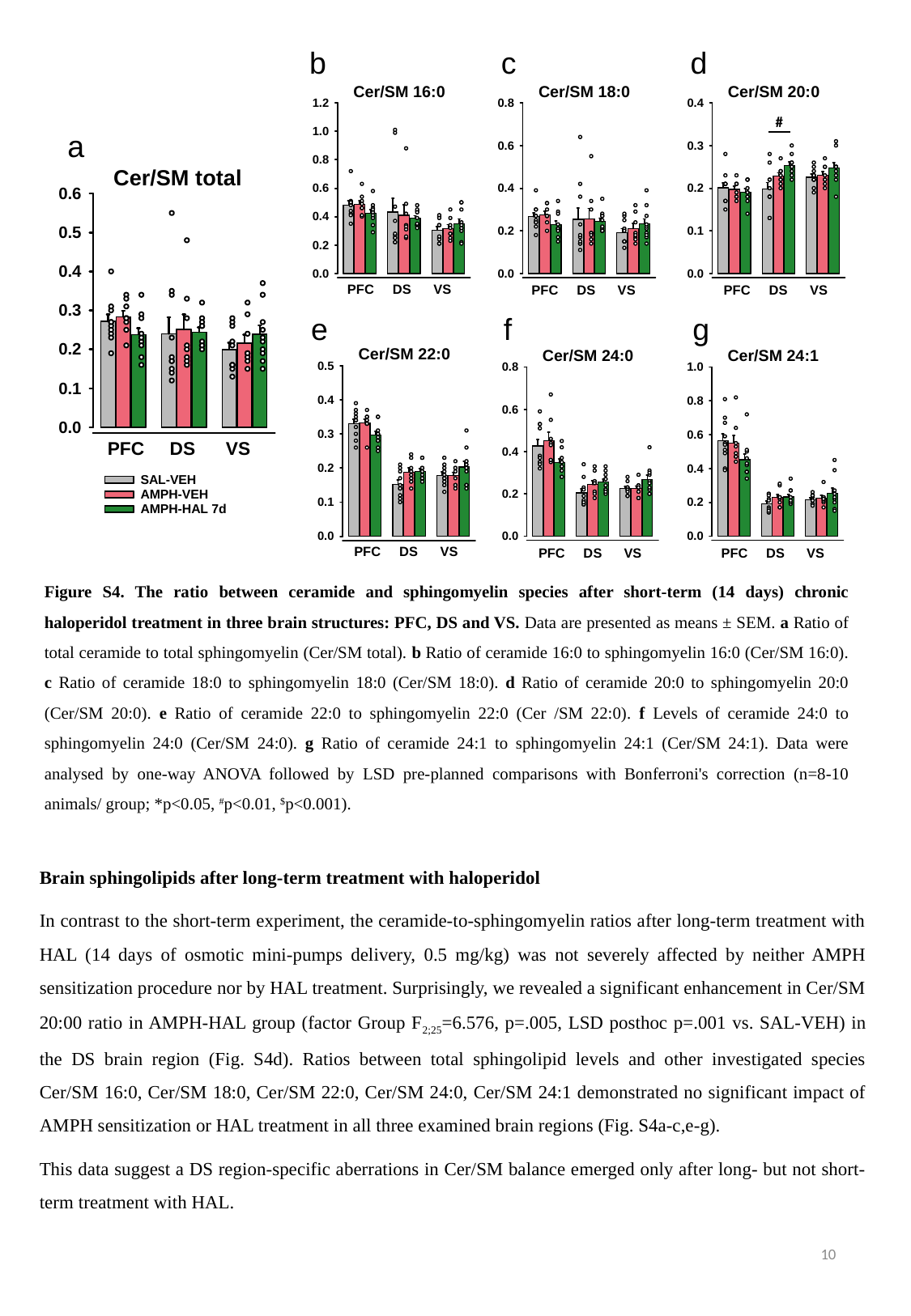

b
c
a
d
e
f
g
#
Figure S4. The ratio between ceramide and sphingomyelin species after short-term (14 days) chronic haloperidol treatment in three brain structures: PFC, DS and VS. Data are presented as means ± SEM. a Ratio of total ceramide to total sphingomyelin (Cer/SM total). b Ratio of ceramide 16:0 to sphingomyelin 16:0 (Cer/SM 16:0). c Ratio of ceramide 18:0 to sphingomyelin 18:0 (Cer/SM 18:0). d Ratio of ceramide 20:0 to sphingomyelin 20:0 (Cer/SM 20:0). e Ratio of ceramide 22:0 to sphingomyelin 22:0 (Cer /SM 22:0). f Levels of ceramide 24:0 to sphingomyelin 24:0 (Cer/SM 24:0). g Ratio of ceramide 24:1 to sphingomyelin 24:1 (Cer/SM 24:1). Data were analysed by one-way ANOVA followed by LSD pre-planned comparisons with Bonferroni's correction (n=8-10 animals/ group; *p<0.05, #p<0.01, $p<0.001).
Brain sphingolipids after long-term treatment with haloperidol
In contrast to the short-term experiment, the ceramide-to-sphingomyelin ratios after long-term treatment with HAL (14 days of osmotic mini-pumps delivery, 0.5 mg/kg) was not severely affected by neither AMPH sensitization procedure nor by HAL treatment. Surprisingly, we revealed a significant enhancement in Cer/SM 20:00 ratio in AMPH-HAL group (factor Group F2;25=6.576, p=.005, LSD posthoc p=.001 vs. SAL-VEH) in the DS brain region (Fig. S4d). Ratios between total sphingolipid levels and other investigated species Cer/SM 16:0, Cer/SM 18:0, Cer/SM 22:0, Cer/SM 24:0, Cer/SM 24:1 demonstrated no significant impact of AMPH sensitization or HAL treatment in all three examined brain regions (Fig. S4a-c,e-g).
This data suggest a DS region-specific aberrations in Cer/SM balance emerged only after long- but not short-term treatment with HAL.
10

## Slide 11
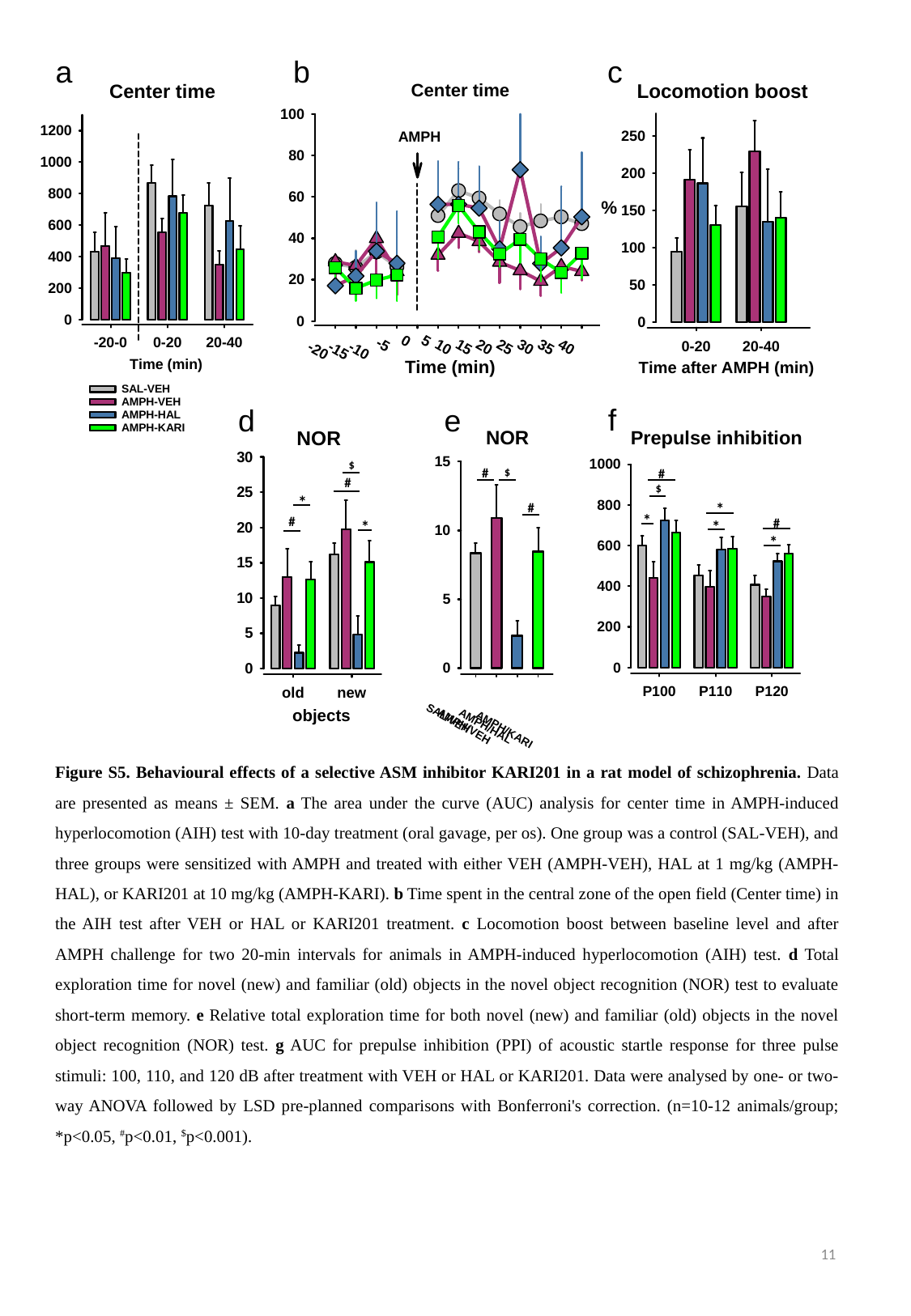

a
b
c
f
e
d
$
#
*
#
*
#
$
#
#
$
*
*
#
*
*
Figure S5. Behavioural effects of a selective ASM inhibitor KARI201 in a rat model of schizophrenia. Data are presented as means ± SEM. a The area under the curve (AUC) analysis for center time in AMPH-induced hyperlocomotion (AIH) test with 10-day treatment (oral gavage, per os). One group was a control (SAL-VEH), and three groups were sensitized with AMPH and treated with either VEH (AMPH-VEH), HAL at 1 mg/kg (AMPH-HAL), or KARI201 at 10 mg/kg (AMPH-KARI). b Time spent in the central zone of the open field (Center time) in the AIH test after VEH or HAL or KARI201 treatment. c Locomotion boost between baseline level and after AMPH challenge for two 20-min intervals for animals in AMPH-induced hyperlocomotion (AIH) test. d Total exploration time for novel (new) and familiar (old) objects in the novel object recognition (NOR) test to evaluate short-term memory. e Relative total exploration time for both novel (new) and familiar (old) objects in the novel object recognition (NOR) test. g AUC for prepulse inhibition (PPI) of acoustic startle response for three pulse stimuli: 100, 110, and 120 dB after treatment with VEH or HAL or KARI201. Data were analysed by one- or two-way ANOVA followed by LSD pre-planned comparisons with Bonferroni's correction. (n=10-12 animals/group; *p<0.05, #p<0.01, $p<0.001).
11

## Slide 12
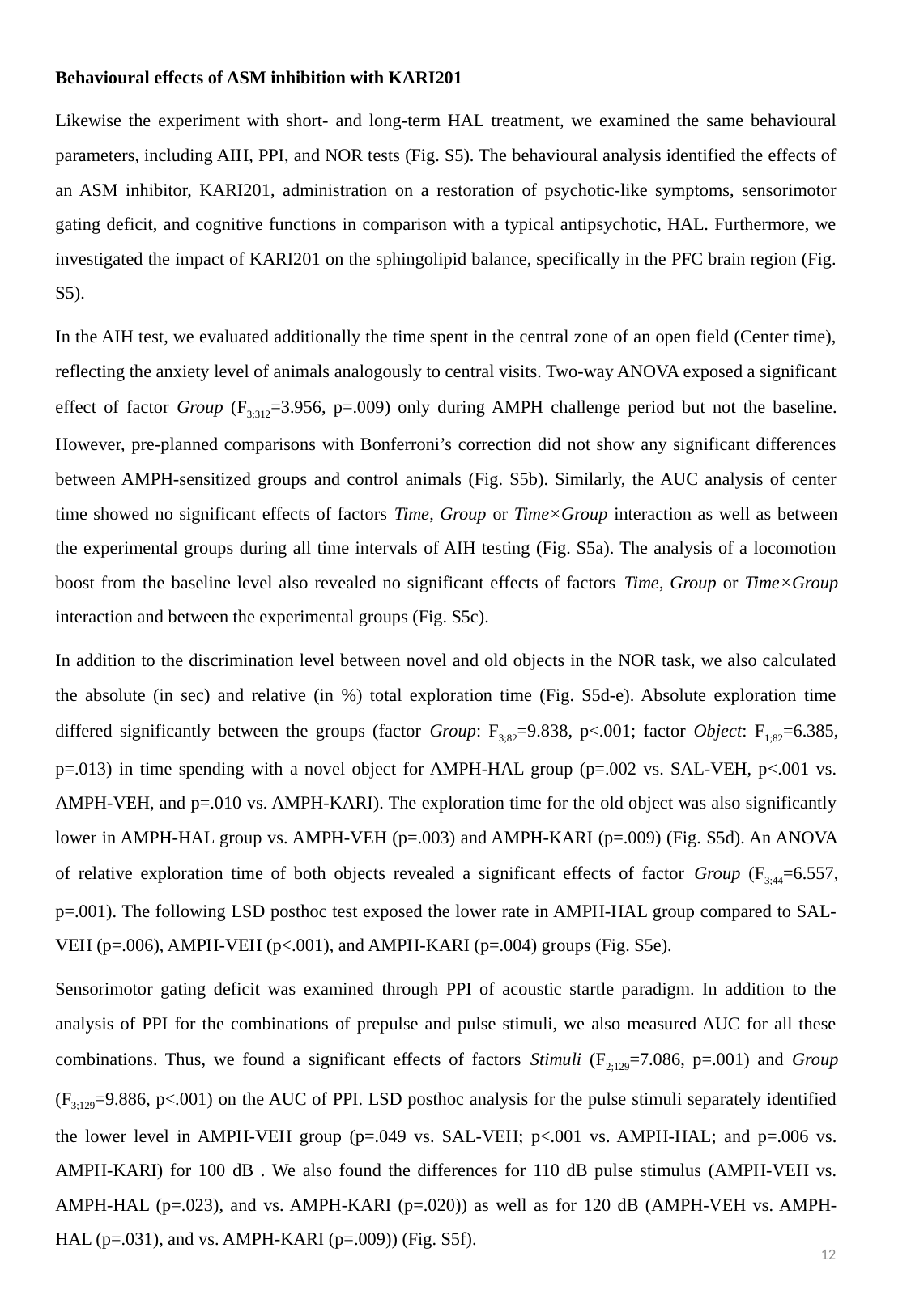

Behavioural effects of ASM inhibition with KARI201
Likewise the experiment with short- and long-term HAL treatment, we examined the same behavioural parameters, including AIH, PPI, and NOR tests (Fig. S5). The behavioural analysis identified the effects of an ASM inhibitor, KARI201, administration on a restoration of psychotic-like symptoms, sensorimotor gating deficit, and cognitive functions in comparison with a typical antipsychotic, HAL. Furthermore, we investigated the impact of KARI201 on the sphingolipid balance, specifically in the PFC brain region (Fig. S5).
In the AIH test, we evaluated additionally the time spent in the central zone of an open field (Center time), reflecting the anxiety level of animals analogously to central visits. Two-way ANOVA exposed a significant effect of factor Group (F3;312=3.956, p=.009) only during AMPH challenge period but not the baseline. However, pre-planned comparisons with Bonferroni’s correction did not show any significant differences between AMPH-sensitized groups and control animals (Fig. S5b). Similarly, the AUC analysis of center time showed no significant effects of factors Time, Group or Time×Group interaction as well as between the experimental groups during all time intervals of AIH testing (Fig. S5a). The analysis of a locomotion boost from the baseline level also revealed no significant effects of factors Time, Group or Time×Group interaction and between the experimental groups (Fig. S5c).
In addition to the discrimination level between novel and old objects in the NOR task, we also calculated the absolute (in sec) and relative (in %) total exploration time (Fig. S5d-e). Absolute exploration time differed significantly between the groups (factor Group: F3;82=9.838, p<.001; factor Object: F1;82=6.385, p=.013) in time spending with a novel object for AMPH-HAL group (p=.002 vs. SAL-VEH, p<.001 vs. AMPH-VEH, and p=.010 vs. AMPH-KARI). The exploration time for the old object was also significantly lower in AMPH-HAL group vs. AMPH-VEH (p=.003) and AMPH-KARI (p=.009) (Fig. S5d). An ANOVA of relative exploration time of both objects revealed a significant effects of factor Group (F3;44=6.557, p=.001). The following LSD posthoc test exposed the lower rate in AMPH-HAL group compared to SAL-VEH (p=.006), AMPH-VEH (p<.001), and AMPH-KARI (p=.004) groups (Fig. S5e).
Sensorimotor gating deficit was examined through PPI of acoustic startle paradigm. In addition to the analysis of PPI for the combinations of prepulse and pulse stimuli, we also measured AUC for all these combinations. Thus, we found a significant effects of factors Stimuli (F2;129=7.086, p=.001) and Group (F3;129=9.886, p<.001) on the AUC of PPI. LSD posthoc analysis for the pulse stimuli separately identified the lower level in AMPH-VEH group (p=.049 vs. SAL-VEH; p<.001 vs. AMPH-HAL; and p=.006 vs. AMPH-KARI) for 100 dB . We also found the differences for 110 dB pulse stimulus (AMPH-VEH vs. AMPH-HAL (p=.023), and vs. AMPH-KARI (p=.020)) as well as for 120 dB (AMPH-VEH vs. AMPH-HAL (p=.031), and vs. AMPH-KARI (p=.009)) (Fig. S5f).
12

## Slide 13
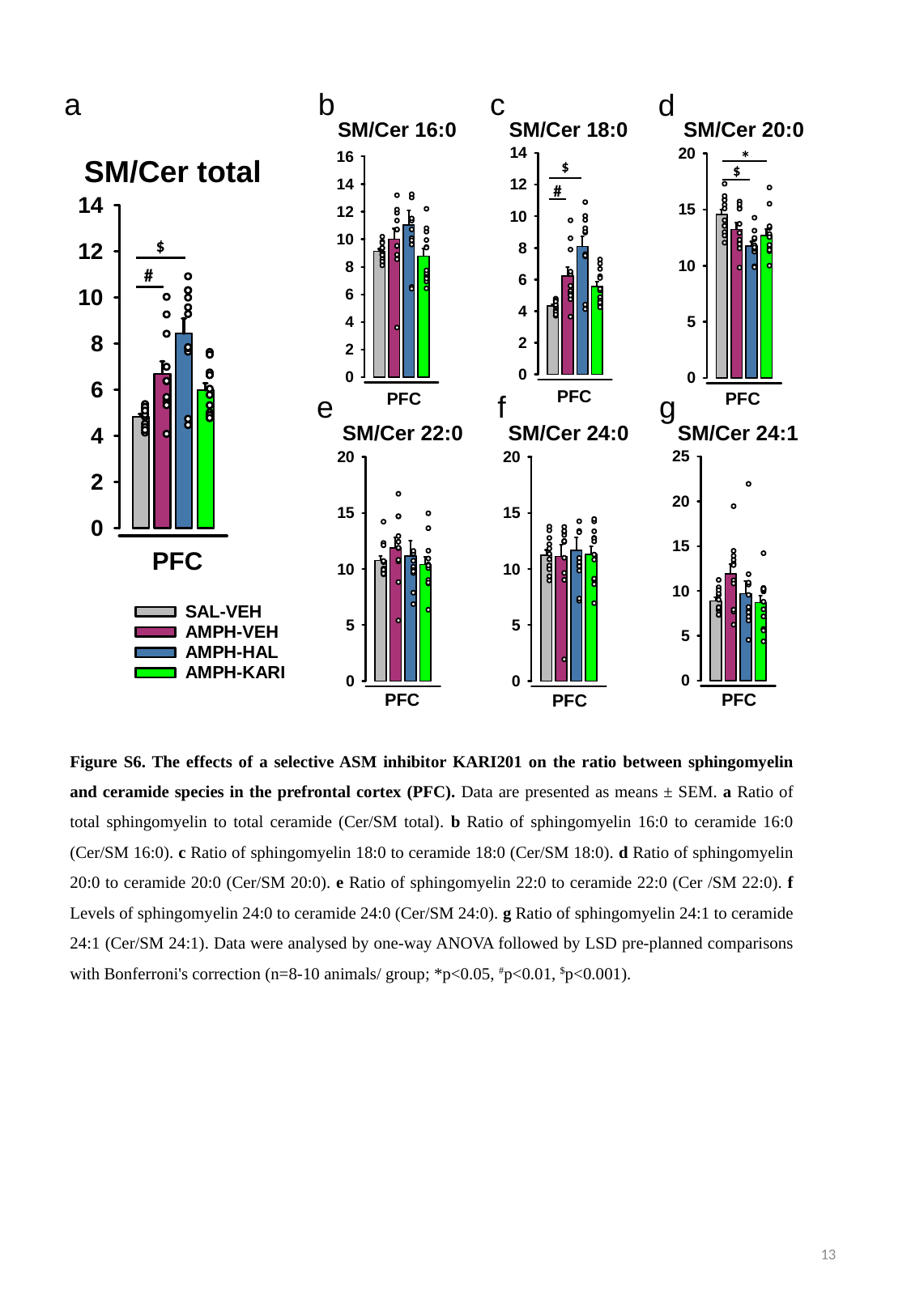

a
b
c
d
e
f
g
$
#
*
$
$
#
Figure S6. The effects of a selective ASM inhibitor KARI201 on the ratio between sphingomyelin and ceramide species in the prefrontal cortex (PFC). Data are presented as means ± SEM. a Ratio of total sphingomyelin to total ceramide (Cer/SM total). b Ratio of sphingomyelin 16:0 to ceramide 16:0 (Cer/SM 16:0). c Ratio of sphingomyelin 18:0 to ceramide 18:0 (Cer/SM 18:0). d Ratio of sphingomyelin 20:0 to ceramide 20:0 (Cer/SM 20:0). e Ratio of sphingomyelin 22:0 to ceramide 22:0 (Cer /SM 22:0). f Levels of sphingomyelin 24:0 to ceramide 24:0 (Cer/SM 24:0). g Ratio of sphingomyelin 24:1 to ceramide 24:1 (Cer/SM 24:1). Data were analysed by one-way ANOVA followed by LSD pre-planned comparisons with Bonferroni's correction (n=8-10 animals/ group; *p<0.05, #p<0.01, $p<0.001).
13

## Slide 14
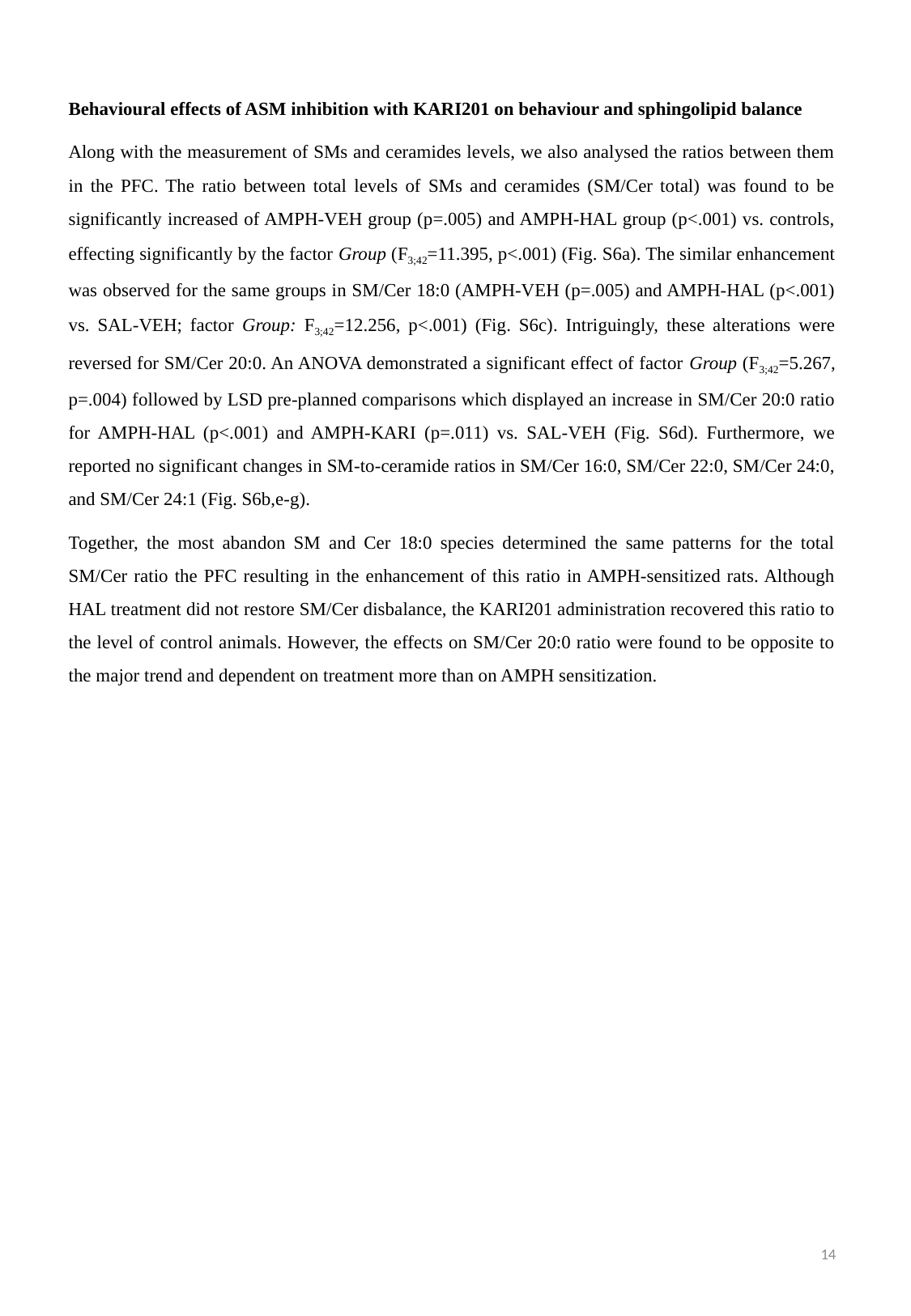

Behavioural effects of ASM inhibition with KARI201 on behaviour and sphingolipid balance
Along with the measurement of SMs and ceramides levels, we also analysed the ratios between them in the PFC. The ratio between total levels of SMs and ceramides (SM/Cer total) was found to be significantly increased of AMPH-VEH group (p=.005) and AMPH-HAL group (p<.001) vs. controls, effecting significantly by the factor Group (F3;42=11.395, p<.001) (Fig. S6a). The similar enhancement was observed for the same groups in SM/Cer 18:0 (AMPH-VEH (p=.005) and AMPH-HAL (p<.001) vs. SAL-VEH; factor Group: F3;42=12.256, p<.001) (Fig. S6c). Intriguingly, these alterations were reversed for SM/Cer 20:0. An ANOVA demonstrated a significant effect of factor Group (F3;42=5.267, p=.004) followed by LSD pre-planned comparisons which displayed an increase in SM/Cer 20:0 ratio for AMPH-HAL (p<.001) and AMPH-KARI (p=.011) vs. SAL-VEH (Fig. S6d). Furthermore, we reported no significant changes in SM-to-ceramide ratios in SM/Cer 16:0, SM/Cer 22:0, SM/Cer 24:0, and SM/Cer 24:1 (Fig. S6b,e-g).
Together, the most abandon SM and Cer 18:0 species determined the same patterns for the total SM/Cer ratio the PFC resulting in the enhancement of this ratio in AMPH-sensitized rats. Although HAL treatment did not restore SM/Cer disbalance, the KARI201 administration recovered this ratio to the level of control animals. However, the effects on SM/Cer 20:0 ratio were found to be opposite to the major trend and dependent on treatment more than on AMPH sensitization.
14

## Slide 15
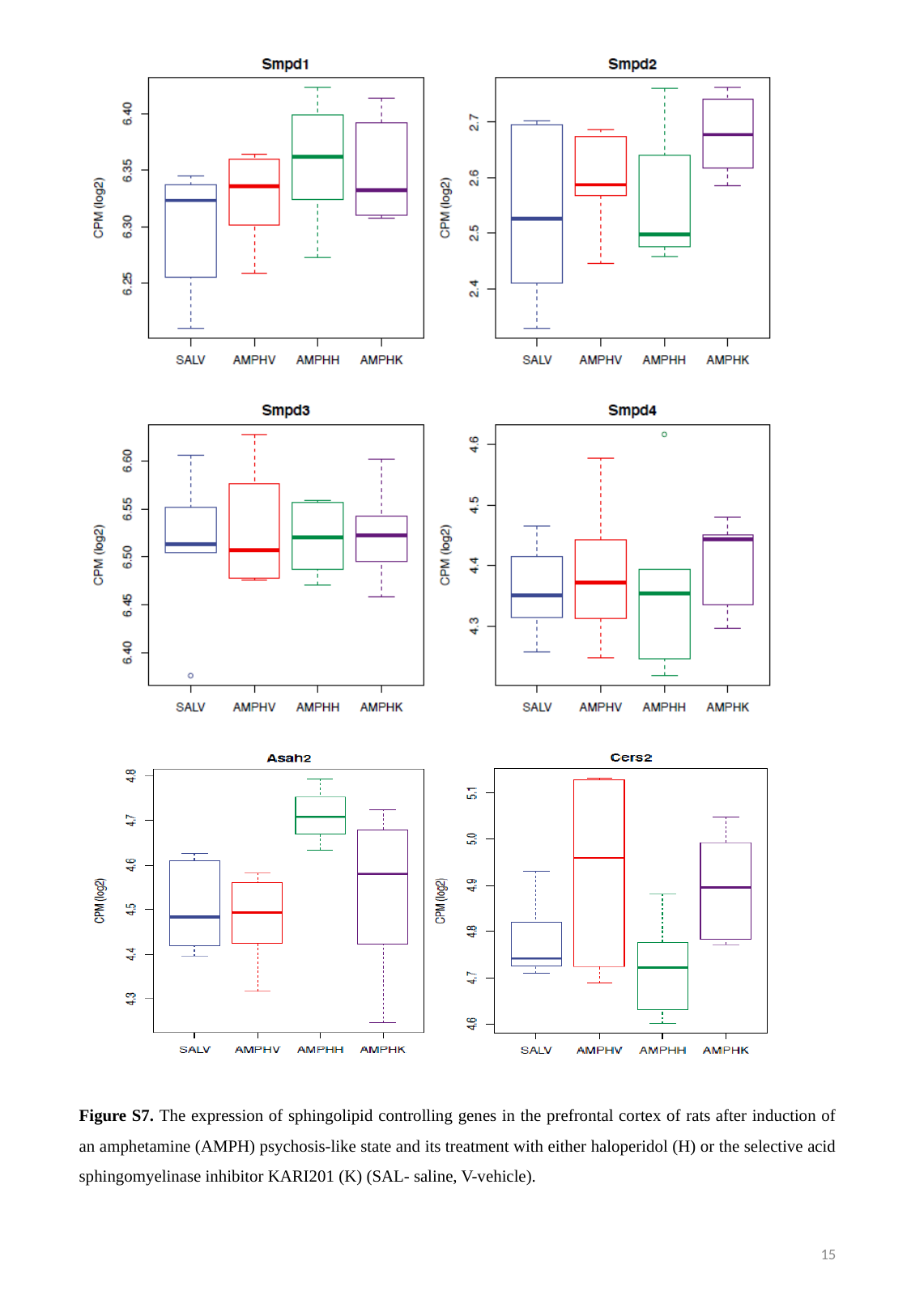

Figure S7. The expression of sphingolipid controlling genes in the prefrontal cortex of rats after induction of an amphetamine (AMPH) psychosis-like state and its treatment with either haloperidol (H) or the selective acid sphingomyelinase inhibitor KARI201 (K) (SAL- saline, V-vehicle).
15
